# Recombination in the Human Pseudoautosomal Region PAR1

**DOI:** 10.1371/journal.pgen.1004503

**Published:** 2014-07-17

**Authors:** Anjali G. Hinch, Nicolas Altemose, Nudrat Noor, Peter Donnelly, Simon R. Myers

**Affiliations:** Wellcome Trust Centre for Human Genetics, Oxford University, Oxford, United Kingdom; University of California San Francisco, United States of America

## Abstract

The pseudoautosomal region (PAR) is a short region of homology between the mammalian X and Y chromosomes, which has undergone rapid evolution. A crossover in the PAR is essential for the proper disjunction of X and Y chromosomes in male meiosis, and PAR deletion results in male sterility. This leads the human PAR with the obligatory crossover, PAR1, to having an exceptionally high male crossover rate, which is 17-fold higher than the genome-wide average. However, the mechanism by which this obligatory crossover occurs remains unknown, as does the fine-scale positioning of crossovers across this region. Recent research in mice has suggested that crossovers in PAR may be mediated independently of the protein PRDM9, which localises virtually all crossovers in the autosomes. To investigate recombination in this region, we construct the most fine-scale genetic map containing directly observed crossovers to date using African-American pedigrees. We leverage recombination rates inferred from the breakdown of linkage disequilibrium in human populations and investigate the signatures of DNA evolution due to recombination. Further, we identify direct PRDM9 binding sites using ChIP-seq in human cells. Using these independent lines of evidence, we show that, in contrast with mouse, PRDM9 does localise peaks of recombination in the human PAR1. We find that recombination is a far more rapid and intense driver of sequence evolution in PAR1 than it is on the autosomes. We also show that PAR1 hotspot activities differ significantly among human populations. Finally, we find evidence that PAR1 hotspot positions have changed between human and chimpanzee, with no evidence of sharing among the hottest hotspots. We anticipate that the genetic maps built and validated in this work will aid research on this vital and fascinating region of the genome.

## Author Summary

Recombination is a fundamental biological process, which shuffles genes between pairs of chromosomes during the production of eggs and sperm. After shuffling, the chromosomes consist of alternating sequences of genes from each parent, where the switches are the result of ‘crossovers’. Recombination is essential for eggs and sperm to receive the correct number of chromosomes, failure in which is an important cause of miscarriage, birth defects and mental retardation. Males have the particular challenge of recombining between the X and Y chromosomes. Unlike the other 22 chromosome pairs, the X and Y chromosomes do not match up, except for a small special region called PAR1, which must host a crossover. We investigate recombination in PAR1 by building a ‘map’ of where it occurs in African-American families. We use a variety of approaches, both analytical and experimental, to demonstrate the role of a protein called PRDM9 in marking crossovers in this region. PRDM9 has previously been shown to position crossovers on the other chromosomes, but a role in PAR1 was unexpected based on research in mice. We also show that the recombination map has changed in the evolutionary history of PAR1, both among human populations, and between human and chimpanzee.

## Introduction

Pseudoautosomal regions (PARs) are segments of sequence homology between the X and Y (or Z and W) chromosomes, which are otherwise non-homologous. Uniquely, PARs are inherited in the same manner as autosomes, while also being partially linked with X-specific and Y-specific loci. They have a critical role in the successful progression of meiosis in mammalian males and in the heterogametic sex in many other plant and animal species [1]–[10]. Correct segregation of chromosomes into gametes during meiosis requires that homologous chromosomes pair up and undergo exchange of chromosomal material known as recombination or ‘crossing over’. In females, the two homologous X chromosomes pair up and can recombine along their entire length [3]. In males, however, pairing and recombination are restricted to the homologous PAR regions. PARs in most mammals are typically a few hundred kilobases to several megabases in length [11]–[14] and make up only a small fraction of the Y chromosome, imposing an extraordinary pressure to achieve recombination in a short genomic segment. Humans have two PARs – PAR1, which is at the tip of the short arm (Xp/Yp) of the sex chromosomes, and PAR2, which is at the tip of the long arm (Xq/Yq). Deletion of PAR1 is associated with total male sterility in humans [5], [15]. Reduced recombination in PAR1 can lead to aneuploid sperm, which can cause X-chromosome monosomy (Turner syndrome) or XXY (Kleinfelter syndrome) in the offspring [7], [16].

In addition to their vital role in fertility, PARs contain genes in all mammals whose sequence has become available so far. The human PARs together contain at least 29 genes, with diverse roles in cell signalling, transcriptional regulation and mitochondrial function [17]. Thus far, SHOX is the only PAR gene which has been definitively associated with a role in normal development [18]. More recently, associations have also been reported with PAR1 loci for schizophrenia and bipolar affective disorder [19], [20].

Studies in viable human sperm and pedigrees have shown that the recombination rate in PAR1 is consistent with one obligatory crossover per male meiosis, accompanied very rarely by a second crossover [2], [21]. PAR1 is approximately 2.7 Mb long, and this leads to PAR1 having a crossover rate 17-fold greater than the genome-wide average, over four times greater than the next most recombinogenic region of comparable size in the genome. In contrast, the female recombination rate in PAR1 is comparable to the genome-wide average [22]–[24]. Human PAR1 shares homology with other mammalian PARs [14], [25]. While PARs in several mammals, including human, horse, cattle, dog and sheep, appear to descend from the same ancestral region [25], the boundary between the PAR and X-specific and Y-specific regions has shifted dramatically, leading to highly variable gene content. The mouse PAR does not share homology with human or any other known mammalian PAR (the ancestral PAR appears to have been lost from the mouse X chromosome). Instead, mice have a different, considerably shorter PAR on the q-arm of the X chromosome, which spans only 700 kb [26], [27].

The second human pseudoautosomal region, PAR2, is much smaller at approximately 330 kb and specific to the human lineage, having likely arisen due to a translocation between the X and Y chromosomes [28]. Crossovers in PAR2 occur rarely, at a rate similar to the genome average, in both sexes [24], suggesting behaviour similar to many autosomal regions. For the rest of this work, we focus our attention on PAR1, the evolutionarily and biologically more significant region.

Despite the critical role of PAR1 in fertility and disease, an understanding of its biology remains highly incomplete. In the reference human genome, the PAR1 sequence is not yet fully assembled, likely because of the exceptionally high GC-content and density of repetitive regions it contains. Since the publication of the X chromosome sequence [17], updates in the human genome release GRCh37 by the Genome Reference Consortium have closed some of the gaps, resulting in a sequence that is 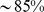 complete. Nevertheless, PAR1 has a far lower density of single nucleotide polymorphisms that are included on genotyping arrays relative to other parts of the genome [29], despite the much shorter extent of linkage disequilibrium (LD) in this region. PAR1 has also largely been neglected in linkage studies and genome-wide association scans, possibly due to the lack of both polymorphism and linkage information. For other mammalian species with otherwise high-quality reference genomes, the PAR sequence is similarly either absent entirely or only partially represented [30].

Even less is known about recombination, which lies at the heart of PAR1 biology. For instance, it is not known how the extraordinarily high rate of recombination in this region is achieved biologically. In the autosomes, recombination clusters into short 1–2 kb segments known as ‘recombination hotspots’, which are flanked by regions with very low recombination rate [31]–[35]. That hotspots are also a feature of PAR1 recombination is implied by the characterisation of a single recombination hotspot within the SHOX gene, which is one of the hottest hotspots measured thus far using high resolution sperm-typing in the genome [36]. However, no further hotspots in PAR1 have yet been characterized. The utility of the fine-scale genetic map based on LD [29] in this region is unclear [37], due to the very rapid breakdown of LD in this region [36]. Other currently available genetic maps for PAR1 that have been built using low resolution sperm-typing and genotyped pedigrees are based on a small number of markers, typically in small sample sizes [2], [21]–[24], [38], [39]. This, along with technical difficulties linked to the relatively small size of PAR1, leads to imprecise estimates, and insufficient resolution to understand the drivers of recombination. The most fine-scale map available to date from directly observed crossovers was built in 28 European ancestry pedigrees genotyped at 22 polymorphic markers in PAR1, corresponding to roughly one marker per 100 kb [24]. The most detailed human pedigree-based map built to date [40], with 15,000 meioses in the Icelandic population, did not include any markers in PAR1. The PAR was also not included in the recent work that built LD-based maps in the chimpanzee [41].

An intriguing study [42] found that pairing of homologous chromosomes occurs significantly later in the PAR than in the autosomes in male mice. They also found that chromosomal axes were significantly longer in the PAR relative to the autosomes during meiosis, and that a different isoform of a key recombination protein (Spo11) is active in this region, implying that distinct recombination machinery may operate here.

The role of another key recombination protein, PRDM9, is also unclear in the PAR. Several lines of evidence have shown recently that PRDM9 positions sites of recombination in human and mice autosomes [43]–[45] by direct binding to recombination hotspots. However, whether PRDM9 plays any role in the male PAR1 is controversial. Recent work in mice [46] has shown that male mice with different *Prdm9* variants have completely different autosomal recombination patterns, yet show similar recombination landscapes in and adjacent to the PAR region. Brick *et al.* [46] have therefore suggested that a mechanism independent of *Prdm9* may be positioning crossovers in the mouse PAR.

In this work, we aim to characterise the patterns of recombination in PAR1 to learn more about the biology of this region, and provide a resource for medical genetics research. We have built the most fine-scale genetic map containing directly identified crossovers to date in this region. This map contains more meioses, and an order of magnitude greater markers than the densest PAR1 map so far [24]. This allows us to analyse recombination in this region at a finer scale than has been possible in the past. It also enables us to assess the accuracy of the LD-based map built using HapMap2 variation data in this region [29]. We use evidence of direct PRDM9 binding in human cells to examine the role of this protein in specifying recombination in PAR1. Finally, we measure the impact and evolution of recombination using observed biases in the allele frequency spectra for different types of mutations due to recombination. We leverage these resources to explore the role of PRDM9, and to infer evolution of recombination in PAR1 within human populations and between human and chimpanzee.

## Results

### A new pedigree-based genetic map for PAR1

We have leveraged the genotype data of 220 markers from 135 African-American families with two or more children to build a new pedigree-based genetic map (Materials and Methods, Text S1, Dataset S1). These data comprise a total of 672 meioses (336 paternal and 336 maternal), in which we could directly detect crossovers between parent and child. Amongst these families, 19 families included genotype data for both parents, and the rest for only one parent. We used methods that we have previously published [47] to detect crossovers in such incomplete pedigrees (Materials and Methods).

**Figure 1:**
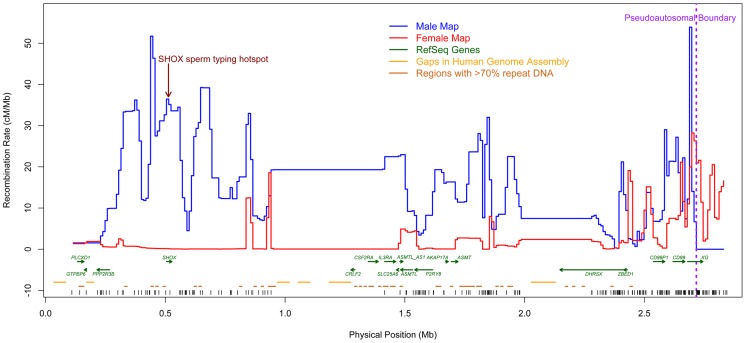
New sex-specific pedigree-based genetic maps (10 kb scale). The male map (blue) shows intense crossover activity throughout PAR1, with particularly high rates towards the telomeric end. Fine-scale variation in rates could not be estimated in two regions (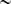
1–1.4 Mb and 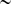
2–2.25 Mb, build 36) due to large unmapped and repetitive sequences and lack of genotyped SNPs. The female map (red) has a low rate through much of PAR1, and a trend of increasing rate towards the pseudoautosomal boundary. Vertical black tick marks show marker positions.

Figure 1 shows the recombination rates estimated in both males and females (Dataset S2). We inferred a total genetic distance of 136 paternal and 18 maternal crossovers in PAR1. The average number of detected events in males (0.4 events per meiosis) is less than the expected number of events (0.5 events per meiosis). This may be due to the paucity of markers in the sub-telomeric 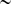
250 kb region of PAR1, which reduces our power to detect crossovers in this region. The number of female events (0.05 events per meiosis) is consistent with previous studies, which have detected between 0.03 to 0.06 events per female meiosis [2],[23],[24],[39]. 126 paternal and 17 maternal crossovers have both endpoints mapping within our region of marker coverage (Datasets S3 and S4). No double crossovers were identified in either sex. Table S1 summarizes the resolution of paternal and maternal events.

We found intense crossover activity throughout PAR1 in males. Only a few loci have an estimated recombination rate that is lower than the genome-wide average rate of approximately 1.2 cM/Mb [47], with little evidence for truly cold regions anywhere in the male PAR1. The previously identified SHOX hotspot [36] is at a peak of male recombination rate (Figure 1). Consistent with the pattern in other chromosomes in males [48], [49], we observed a significant trend of reduction in rate away from the telomere (Tables S2 and S3). In contrast, in females, we observed the lowest rate near the telomeres and the highest rate near the pseudoautosomal boundary, and the differences are significant (Tables S2 and S4). The male rate increases somewhat in the vicinity of the pseudoautosomal boundary (Figure S1).

In the rest of this work, we use these maps to validate the sex-averaged HapMap2 LD-based map, and to learn about the biological drivers of recombination in this region.

### Validation of the HapMap2 LD-based map in PAR1

The HapMap2 LD-based map is the most fine-scaled map currently available for PAR1 with rates inferred between nearly 1,400 markers [29]. This map was built using genotypes from unrelated individuals from three HapMap Phase II populations – European ancestry individuals from Utah (CEU), Yoruba individuals from West Africa (YRI) and Asian individuals from China and Japan (JPT+CHB). Maps specific to each of these populations have also been built, and are referred to as the CEU, YRI and JPT+CHB maps respectively. LD-based maps are built by inferring recombination from the observed breakdown of linkage disequilibrium between markers, and capture information from tens of thousands of meioses over thousands of generations of human history. They have been found to be reliable estimates of historical recombination rates in the autosomes, in comparisons with numerous pedigree-based maps and high-resolution sperm-typing experiments [40], [50].

In PAR1, however, the use of LD-based maps raises special concerns specific to this region. The first concern is that rate estimates in the map may be biased downwards, which we call ‘saturation’ of rates. This is because recombination is inferred from the breakdown of LD between markers. If the recombination rate is very high, nearby markers may segregate practically independently. Since further recombination cannot meaningfully reduce the LD in this situation, it may not be possible to infer any difference between very high rates, in practice. The second concern is that the role of selection in PAR1, to ensure male fertility, is unknown, and strong selection might bias the estimation of rates. Therefore, it is vital to empirically confirm the map using a resource which is not influenced by these factors. Finally, LD-based maps are sex-averaged. Since male recombination in PAR1 is of particular interest, we also assess how informative this map is for male recombination.

**Figure 2:**
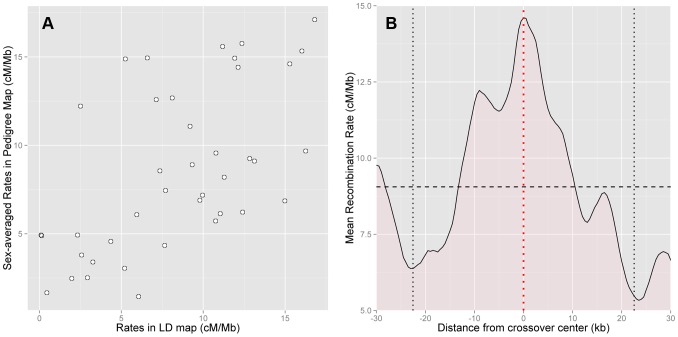
Concordance between pedigree-based and HapMap2 population-averaged LD-based estimates of recombination. (A) Comparison of sex-averaged pedigree rates and LD-based rates at the 50 kb scale shows high correlation (Spearman's 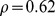, 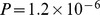). Recombination in PAR1 is dominated by crossovers in males, and the LD-based map is informative about male recombination (Spearman's 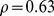, 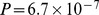). (B) Rates in the LD-based map (5 kb scale, at 500 bp intervals) averaged over the best-resolved 10% of paternal crossovers (n = 12, resolution 13 kb–45 kb, maximum extent shown by vertical black dotted lines), centred such that they all have their midpoint at 0 (red dotted line). PAR1-wide average LD-based rate of 9.06 cM/Mb is shown with the horizontal black dashed line. The LD-based map has a rate significantly elevated above the average rate at crossover midpoints (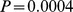).

To check the accuracy of the HapMap2 population-averaged LD-based map, we compared it with the sex-averaged rates from our pedigree map, and found good agreement between the two maps (Figure 2a). The correlation between the maps is high despite considerable statistical uncertainty in the estimation of the pedigree-based map (Spearman's 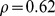 at 50 kb scale, 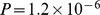). Further, there is no evidence of downward bias among high rate regions in the LD-based map (Figure 2a). This suggests that saturation of rates is not a significant concern.

Approximately 90% of the historical crossover events in PAR1, which influence LD patterns in the region, are expected to have occurred in males. Therefore, we anticipate that the LD-based maps are dominated by male recombination. This is confirmed by the correlation of the male-specific pedigree-based map with the population-averaged LD-based map (Spearman's 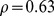 at 50 kb, 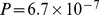), which is approximately the same as that of the sex-averaged map.

Next, we assessed how accurately hotspots in the HapMap2 population-averaged LD-based map are localised by comparing them with the location of crossovers in the pedigrees. Specifically, we calculated the average rate around the centres of the best-resolved 10% of crossovers in pedigree fathers, whose resolution ranged from 13 kb to 45 kb. We found that the LD-based map has a clear peak precisely centred at the sites of crossovers (Figure 2b). This rate elevation to 14.6 cM/Mb above the average rate of 9.1 cM/Mb is significant (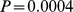, 5000 bootstrap iterations over the crossovers). We conclude that the LD-based map predicts rate peaks at crossover sites in African-American fathers.

Recombination in African Americans has previously been modelled using a linear combination of the CEU and YRI maps in the autosomes [47], [51]. The ratio of the two maps (79%:21%) for the best linear combination of the two maps was similar to the average underlying ancestry proportions (80%:20%) in the admixed individuals [51]. We applied the same approach to the PAR1 map of our African-African fathers. If the CEU, YRI and the pedigree-based maps in PAR1 are the same, we would expect the best linear combination to be an equal 0.5∶0.5 weighting of the CEU and YRI maps, while differences between the maps should result in a higher YRI contribution. We found that, at the 10 kb scale, the best map is a weighted average of 70% (s.e. = 8%) YRI map and 30% (s.e. = 8%) CEU map. It is significantly different from an equal weighting of the two maps (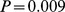). We also performed a model-free analysis by bootstrapping over the pedigree fathers, and calculating the mean squared difference of each bootstrap map with the CEU and YRI maps. We found that the YRI map is significantly more similar to the pedigree map than the CEU map (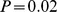). This indicates that the LD-based approach has power to detect differences in the populations, and also suggests that the two populations have systematic differences in the first place. Although this analysis is suggestive, departure from the assumption of equal error in the CEU and YRI maps may also explain the results, in particular if the CEU map is less informative than the YRI map. However, other forms of evidence also support a population difference, but do not support lower error in the YRI map, as shown below.

These analyses show that the LD-based approach is reliable, accurate, and informative specifically about male recombination. This allows us to use both the pedigree-based and the LD-based maps in the rest of this work.

### The protein PRDM9 positions recombination in PAR1 via binding to specific DNA motifs

Recent work has shown that the chromatin-modifying protein PRDM9 positions the sites of practically all recombination hotspots in human and mouse autosomes [43]–[45]. PRDM9 contains a domain of C2H2 zinc fingers, which is remarkable for being the fastest evolving zinc finger domain in the genome [52]. There are, for example, no PRDM9 zinc fingers known to be present in more than one of the great ape species [44], and dozens of different zinc finger arrays have been characterized in humans [53]. Changes in the PRDM9 zinc-finger array are accompanied by shifts in the recombination landscape: multiple groups have shown that nearly all autosomal recombination is controlled by PRDM9 [46], [47].

A previous study [54] analysed over 30,000 LD-based hotspots and identified a 13-bp motif CCnCCnTnnCCnC (where ‘n’ may be any of the four bases) that marks approximately 40% of human hotspots. In the autosomes, only a fraction of the instances of this motif become hotspots [54]. More recently, the role of this motif has been understood through the realization that certain alleles of PRDM9, including the most common human allele, called allele A, bind this motif via the PRDM9 zinc finger array [43]. It has been shown that individuals with PRDM9 alleles binding to significantly different motifs have no shared autosomal hotspots [46], [47]. However, as discussed above, recent research suggests that *Prdm9* may not have a role in specifying recombination in the PAR in mice [46].

**Figure 3:**
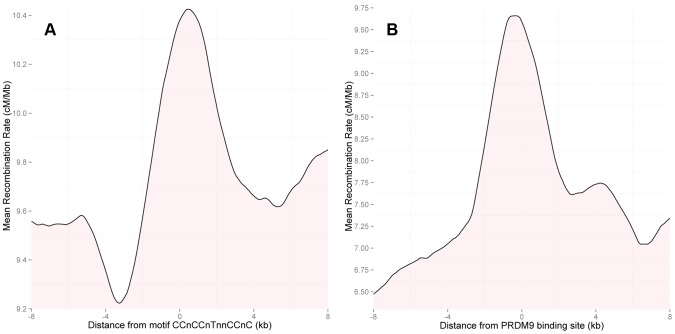
(A) Rate in the HapMap2 population-averaged LD-based map around instances of the 13-bp motif CCnCCnTnnCCnC in PAR1. Motifs in regions with an average SNP density of greater than one SNP per 2 kb in the surrounding 50 kb were included; clusters of motifs within 150 bp of one another were thinned to the most central motif. The plot shows 2 kb averaging, at 100 bp intervals. Motif positions show a strong local increase in recombination rate in the LD-based map. (B) As (A), but showing rates around ChIP-seq binding locations of the PRDM9 reference allele (B allele). In the event of more than one peak within 10 kb, only the most strongly signalled peak was included. ChIP-seq binding peaks of PRDM9 show a stronger rate increase in the LD-based map above local background rate than 13-bp motif sites.

To investigate whether PRDM9 is activating recombination in the human PAR1, we examined the recombination rate near exact matches to the motif CCnCCnTnnCCnC. We observed a sharp increase in the rate in the HapMap2 population-averaged LD-based map in the immediate vicinity of the motif (Figure 3a), comparable in magnitude to the increase observed previously in the autosomes [44]. In the autosomes, the likelihood of the motif resulting in a hotspot is several times greater in THE1A/B and L2 repeat elements, relative to other occurrences of the motif. While there are no copies of the motif within THE1A/B elements currently assembled in PAR1, there are 4 copies of L2 elements that contain the motif and around which rates could be measured. The recombination rate around these elements is nearly twice the regional rate (Figure S2), and the rate elevation is over 5 times greater as compared with other occurrences of the motif in PAR1. This weakly supports a greater increase in rate in such elements, consistent with the autosomes. Moreover, because PRDM9 binds the motif, the observation of a highly localized crossover rate increase around the motif conclusively demonstrates a role for this protein in PAR1.

While the bioinformatically predicted and inferred motif CCnCCnTnnCCnC narrows down the scope of PRDM9 binding sites in the genome, the relationship between motifs, binding sites and recombination hotspots is not perfect [53]–[55]. For example, zinc-finger proteins can bind DNA in a large variety of possible configurations, which are not fully understood [53], [56]. As a result, DNA sequences that appear unlikely to be bound *in silico* have been shown to bind *in vitro* [57].

To address this for PAR1, we measured PRDM9 binding experimentally via chromatin immunoprecipitation followed by high-throughput sequencing (ChIP-seq) in human cells (Materials and Methods). Specifically, we measured the binding of PRDM9 allele B, which is the human reference allele, and is predicted to have binding properties similar to PRDM9 allele A [43]. We identified 185 PRDM9 binding peaks in PAR1 (Materials and Methods). The LD-based map shows a sharp increase in rates at these peaks (Figure 3b), directly connecting PRDM9 binding with local recombination rate increases in this region. Notably, the rate elevation is more than two-fold the increase observed for the 13-bp motif alone (Figure 3a). This is consistent with the fact that the *PRDM9* binding peaks constitute direct evidence of binding. Further, PAR1 peaks containing close matches to the motif are more strongly signalled and show a stronger increase in the LD-based rate than peaks without the motif (Figure S3), suggesting that strength of PRDM9 binding is correlated with recombination rate.

Finally, we report an intriguing characteristic of the binding peaks in PAR1. Approximately 42% of PAR1 peaks contain close matches to the motif, which is consistent with the expected number of hotspots containing the motif in the autosomes [54]. Nearly a fifth of the peaks contain 5 or more and 5% of the peaks contain 12 or more copies of the motif. Many of these peaks are composed of low complexity minisatellite-like tandem repeat structures of periodicity varying from 4 bases to 101 bases. Other tandem repeats containing matches to the PRDM9 binding motif have been observed to be unstable and biased towards gain of repeat units in the human male germline [58]–[60], and this might present an interesting counterbalancing mechanism to the loss of motifs due to preferred transmission of recombination-suppression alleles.

### Evolution of recombination and PRDM9 binding sites in PAR1 within the human lineage

The PRDM9 zinc finger array is highly variable in humans, with around 40 different alleles that have been identified so far [53], [61]. Alleles can be grouped into 5 categories, depending on the number of bases at which their bioinformatically predicted binding sequence matches the 13-bp motif CCnCCnTnnCCnC (known alleles match between 4 and 8 out of the 8 non-degenerate bases in the motif). These categories have differing allele frequencies across different human populations [53]. Variants predicted to match the 13-bp motif exactly (8/8 match) are predominant in Europeans (91%) and Asians (also approximately 91%), but occurred at only about 58% frequency in an African sample [53]. In Africans, approximately 35% of PRDM9 alleles (5/8 match) are strongly predicted not to bind the 13-bp motif [47], [53]. This leads to Africans having reduced activity, on average, in the hotspots activated by alleles most common in Europeans. Instead, they are recombinationally active at novel hotspots not active in most Europeans [47], [53].

As shown in a previous section, African-American pedigree fathers have a significantly greater usage of the African (YRI) map than the European (CEU) map (*P* = 0.009). This suggests that recombination has evolved within the human lineage in PAR1, in a manner similar to the evolution observed in the autosomes.

**Figure 4:**
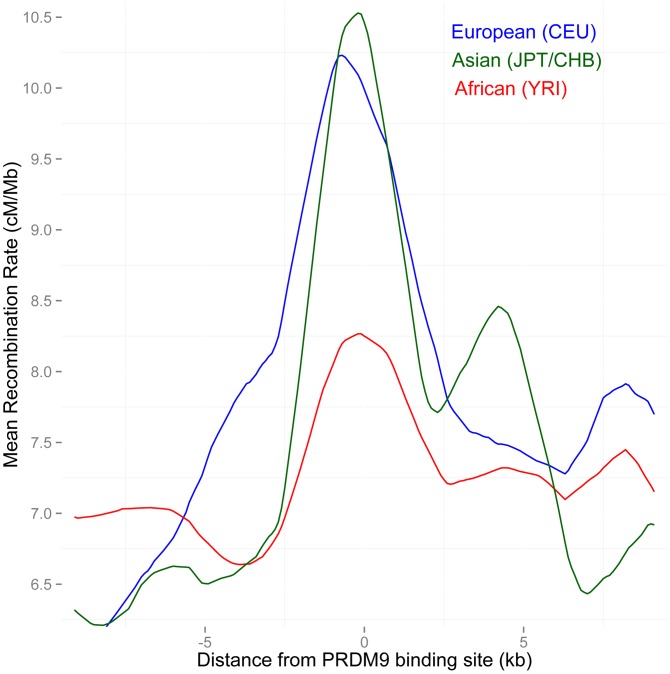
Separate LD-based recombination rates in PAR1 in three human continental groups, around the binding sites of the PRDM9 B allele. The B and other alleles predicted to bind similar motifs predominate in Europe and East Asia (91% frequency), but not in Africa (58% frequency). In PAR1, the recombination pattern is consistent with being activated by PRDM9, as both Asian and European populations show a much stronger increase in rate at these binding sites than Africans (*P* = 0.002 African/Asian, *P* = 0.02 African/European).

To test this further, we examined rates across PAR1 in three population-specific maps, the European (CEU), African (YRI), East Asian (JPT+CHB) LD-based maps at the ChIP-seq binding sites of allele B, which is predicted to bind the 13-bp motif. As expected, the increase in rate in both the Asian and European maps near the binding sites is greater than that in the African map (*P* = 0.002 and 0.02 respectively) (Figure 4). This suggests that the CEU map is unlikely to be systematically less informative than the YRI map. As expected from the similar allele frequencies of the variants matching the 13-bp motif in Europe and Asia, there is no significant difference between the increase in rate in the European and Asian maps near B-allele binding sites.

### Recombination is inferred from strong bias towards GC bases in the sequence evolution of PAR1, and implicates PRDM9 as a marker of recombination in this region

Programmed double-strand breaks leading to recombination may be resolved in one of two ways, as crossovers, which involve reciprocal exchange of chromosomal material, or as non-crossovers, which do not [62], [63]. Both of these outcomes are accompanied by the non-reciprocal copying of a tract of DNA from one participating chromosome to another, known as *gene conversion* [63]. This process is said to be biased if one of the two chromosomes is systematically more likely to be used as the template for copying than the other chromosome, and this phenomenon is referred to as *biased gene conversion* (BGC). Several types of bias have been observed in different eukaryotes [64]–[68], among which is a bias favouring GC over AT alleles, referred to as GC-biased gene conversion (gcBGC) [66], [67], [69], [70]. gcBGC tends to increase the frequency of GC bases in the pool of gametes relative to 50%:50% Mendelian segregation.

A broad range of evidence, across several eukaryotic taxa, indicates that bias towards GC bases is associated with recombination[41], [66], [67], [69]–[74]. A detailed study of gene conversion tracts in yeast directly demonstrated the over-transmission of GC alleles [66], and a recent re-analysis of the data indicates that the bias may be specific to recombination events that are resolved as crossovers [70]. Patterns of variation both within and between species have shown a skew towards GC alleles that correlates strongly with recombination rates in primates, and particularly with recombination hotspots [41], [73]–[76]. The mouse gene *Fxy* presents a particularly striking case study, indicating that GC-bias may operate in the mouse PAR as well. This gene has translocated from the non-recombining part of the mouse Y-chromosome to its PAR within the last 3 million years [77]. This translocation has been followed by an extremely rapid increase in GC content at both coding and non-coding sites [69], [77]. While the molecular mechanisms causing gcBGC are not well understood, recombination is the only known force producing this bias [67], [70].

We investigated whether such a bias is observed in the human PAR1, both in the frequency of segregating sites and for the fixation of alleles leading to substitutions between human and chimpanzee. We reasoned that such a bias, if present, should act as an indirect marker of sites undergoing recombination in the two species, even in the absence of direct evidence on recombination sites in PAR1 in the chimpanzee. We investigated these patterns in (relatively) hot and cold regions of PAR1, and around copies of the 13-bp motif CCnCCnTnnCCnC, which marks peaks of recombination in PAR1 as shown above. Finally, we compared the distribution of GC-altering substitutions between human and chimpanzee to understand the evolution of recombination hotspots between the two species.

PAR1 in humans has a far higher GC content than the rest of the X chromosome (48% vs 39%) [18]. This is also true in chimpanzee (*Pan troglodytes*), which again has 48% GC content in the PAR. We used 1000 Genomes data [78] in PAR1 to obtain a set of sites segregating in human populations at a minor allele frequency of at least 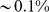. We restricted the set to those sites where the chimpanzee allele is known, and assigned the chimpanzee allele to be the ancestral allelic state. Further, we filtered out all sites where either the ancestral or derived allele is part of a CpG dinucleotide to reduce noise due to repeat mutations resulting from the deamination of methylated CpGs.

**Figure 5:**
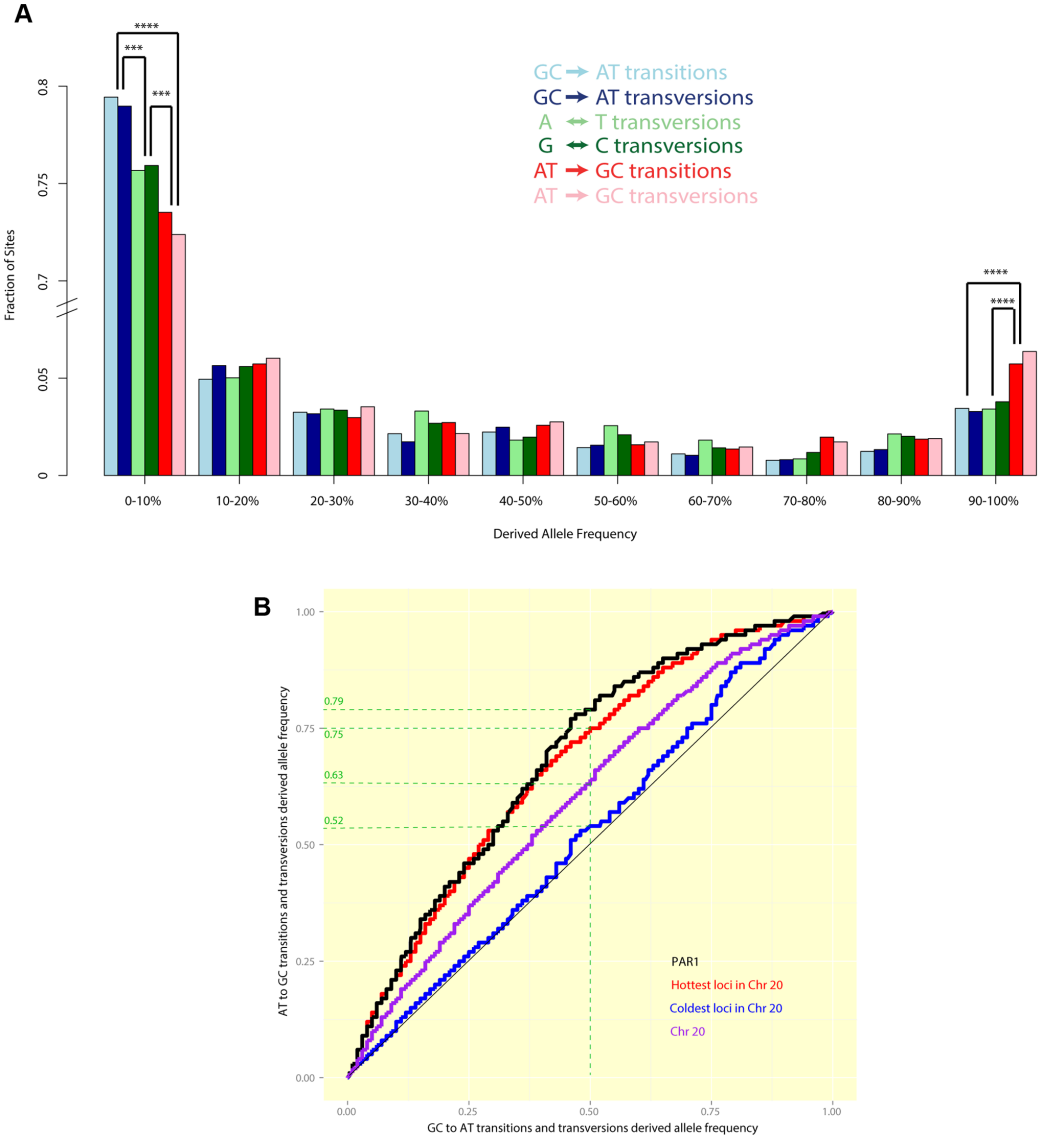
(A) A comparison of allele frequency spectra of different mutation types segregating in humans in PAR1. A significantly greater fraction of GC-increasing mutations have allele frequencies >90%, while a significantly greater fraction of GC-reducing mutations have allele frequencies <10%. Differences with 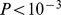 are marked (3 asterisks indicate 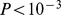 while four indicate 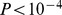). (B) Quantile-quantile plots show the difference between the allele frequency spectra of GC
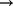
AT mutations (x-axis) and AT
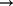
GC mutations (y-axis) in PAR1 and Chr 20. If both types of mutation had the same allele frequency spectrum, we would expect to see a straight line. Points above the diagonal indicate that AT
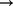
GC mutations are at higher frequencies than GC
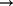
AT mutations, while points below the diagonal show the opposite trend. For example, the green dashed guide lines show that, in PAR1 (black) the same proportion of AT
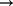
GC sites has allele frequencies 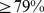 as GC
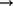
AT sites with allele frequencies 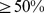. This bias towards higher allele frequencies for AT
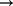
GC mutations is significant (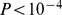). It is comparable to the bias in the hottest 15% of loci, 1 kb in size, in Chr 20 (red), which have a sex-averaged rate comparable to that of PAR1 as a whole (8.2 cM/Mb). The coldest 15% of loci in Chr 20 (average rate 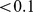 cM/Mb) do not show a significant elevation of GC allele frequencies (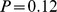).

Figure 5a shows the allele frequency distribution of all six classes of segregating sites in PAR1: GC
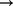
AT transitions and transversions (which reduce GC content), AT
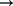
GC transitions and transversions (which increase GC content), and A
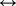
T and C
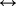
G transversions (which leave GC content unchanged). We observed that mutations that increase GC content are enriched at the top-end of the frequency spectrum, while mutations that decrease GC content are more concentrated at the bottom end of the frequency spectrum. Specifically, we noted that a significantly greater proportion of mutations that increase GC content segregate with allele frequency 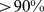 than GC-reducing and GC-neutral mutations (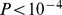). Correspondingly, GC-increasing mutations are less likely to segregate with allele frequency 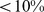 than GC-neutral mutations (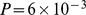), while the opposite is true of GC-decreasing mutations (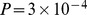). Among GC-increasing (or GC-decreasing) mutations, no significant difference was observed between transitions and transversions at any allele frequency. This is consistent with the expectation of gcBGC in the autosomes, however the ‘U-shape’ of the distribution is much more pronounced in PAR1 than in Chr 20, which is the autosome with the highest chromosome-wide recombination rate in the human genome [47] (Figure S4).

Figure 5b shows a comparison of the full allele frequency spectra of GC
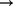
AT and AT
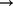
GC mutations in the form of a quantile-quantile plot (details in figure legend). AT
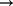
GC mutations in PAR1 segregate at significantly higher allele frequencies, on average, than GC
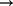
AT mutations (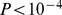). We compared this with the pattern in Chr 20. The hottest 15% of loci of size 1 kb in Chr 20 have an average rate of 8.2 cM/Mb, which is comparable to the sex-averaged rate in PAR1. AT
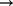
GC mutations segregate at higher frequencies than GC
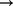
AT mutations at these loci, to an extent similar to PAR1 (Figure 5b). This suggests that the mechanism causing the bias towards GC alleles operates similarly in PAR1 as it does in the autosomes, and that the strength of gcBGC may be similar in males and females. The coldest 15% of Chr 20, with an average rate of 0.02 cM/Mb, does not show a significant excess of GC-mutations, confirming that recombination is causing the bias towards GC-mutations. We note that a quantitative relationship between recombination rate and gcBGC is also confirmed in PAR1, where we observe that the more telomeric 200–700 kb of the PAR has a significantly stronger gcBGC effect than the 500 kb nearest the pseudoautosomal boundary (Figure S5), consistent with its higher average recombination rate.

**Figure 6:**
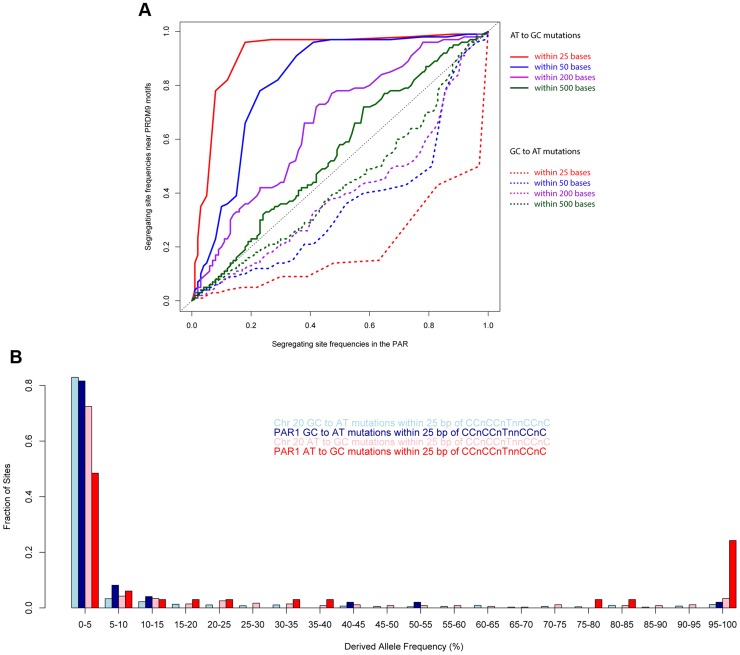
Allele frequencies near the PRDM9 binding motif show the strong influence of recombination. (A) Quantile-quantile plots comparing AT
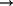
GC and GC
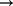
AT allele frequencies in the vicinity of the motif CCnCCnTnnCCnC relative to the rest of PAR1 in human populations. The elevation of GC allele frequencies and suppression of AT alleles is extremely powerful closest to the motif, and drops off rapidly with distance away from it. (B) A comparison of the allele frequency spectra of different mutation types within 25 bp of copies of the motif CCnCCnTnnCCnC in PAR1 and Chr 20 shows an extreme skew towards GC bases in PAR1.

We examined the role of PRDM9 by examining the allele frequency distributions of GC
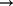
AT and AT
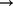
GC mutations near the motif CCnCCnTnnCCnC. A prediction of the recombination-driven gcBGC hypothesis is that the effect should be strongest near recombination hotspots. As shown in Figure 6a, we compared the allele frequency spectrum of AT
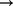
GC mutations *near the motif relative to that class of mutations in PAR1 as a whole*. We observed that the elevation of the allele frequencies of GC mutations near the motif is extreme, and far stronger, over and above the rest of PAR1 (which already shows a strong GC bias). The signal is local to the motif, and weakens rapidly with distance away from it (It is significantly stronger within 25 bases of copies of the motif relative to PAR as a whole (*P* = 0.008), and also relative to within 500 bases of copies of the motif (*P* = 0.01)). The lowering of allele frequencies of AT mutations is also extremely strong near the motif relative to the rest of PAR1. The effect is strongest within 25 bp of the motif, and weakens with distance from it (*P* = 0.02 relative to PAR as a whole).

We expect that, due to the much higher male recombination rate in PAR1, the GC-bias in PAR1 is driven mainly by male recombination. We confirmed this by comparing two regions with opposite trends in male and female recombination rates (Figure S5). Therefore, the patterns of GC-bias near the motif and throughout PAR1 cannot be explained by female recombination alone.

Brick *et al.* [46] have proposed that, in the mouse PAR, there is a cline of PRDM9 activity – with no activity in the most telomeric region and increasing activity with distance away from the telomere. We found no evidence for such a trend in humans. In the human PAR1, the elevation of GC allele frequencies and suppression of AT allele frequencies near the PRDM9 motif are at least as strong in the most telomeric region of PAR1 where rates could be estimated (200 kb–700 kb), as it is near the pseudoautosomal boundary (Figure S6). This region excludes the most telomeric 200 kb, where rates could not be reliably estimated due to lack of markers.

We examined whether gcBGC has an effect on substitution rates in PAR1. Figure 5a suggests that a segregating GC variant in PAR1 is about 1.9 times more likely to be near fixation as a segregating AT variant. To estimate bias in the overall rate of fixation of AT
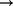
GC and GC
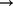
AT variants while accounting for differences in mutation rates [79], we count segregating sites of each type using only derived alleles with allele frequencies between 95% and 100%. We found that, for Chr 20, the higher rate of being near fixation of individual GC alleles is offset by the greater number of GC
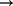
AT segregating sites (bias estimate  = 0.97). However, in PAR1, the number of GC bases near fixation exceeds that of AT bases by almost 20% (bias estimate  = 1.19, *P* = 0.05). We note that this estimate is conservative since a subset of variants will have the wrong ancestral allele assigned due to polymorphism or errors in the chimpanzee (assuming that AT
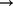
GC and GC
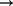
AT mutations are equally likely to have the wrong ancestral allele).

Within 25 bases of the 13-bp motif CCnCCnTnnCCnC (Figure 6b), the fixation bias towards GC is extremely high – 8 times as many GC bases are near fixation as AT bases (bias estimate  = 8.0 and *P* = 0.003, and compared with Chr 20 bias estimate  = 1.15). Another way to estimate the fixation bias close to the motif, in a conservative way, is to model the allele frequency distribution of derived GC alleles as a mixture of the PAR-wide allele frequency distribution of GC alleles, and a perfectly symmetric U-shaped distribution representing a situation where derived alleles are either newly arisen or completely fixed. Such an analysis indicates that 28.4% of motifs in the PAR are extremely active. This contrasts with an estimated 3% of motifs in Chr 20, which is consistent with previous autosomal estimates [54]. This suggests that the higher recombination rate in PAR1 may be supported by nearly an order of magnitude greater availability of motifs for binding via PRDM9.

### Recombination inferred from human and chimpanzee PAR1 sequence changes shows that recombination hotspots have evolved differently in the two species

In the section above, we showed that recombination in PAR1 strongly accelerates the fixation of AT
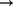
GC mutations relative to GC
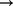
AT mutations. While the overall GC content is similar in the PAR in human and chimpanzee, we ask if the location of substitutions differs in the two species. A region that is a hotspot in one species but not in another is likely to accumulate more GC-substitutions in the first species. In other words, if two species are significantly different in their hotspot landscape, we would expect to see a corresponding signature in the location of their respective GC substitutions.

We test this hypothesis by comparing human and chimpanzee PAR sequence. While no fine-scale genetic map is available for the chimpanzee PAR, we compare substitutions in the two species in regions which are hotspots in humans. Specifically, we consider substitutions in syntenic regions using a human-chimpanzee sequence alignment (Materials and Methods). If hotspots are the same in both species, we expect to see comparable numbers of 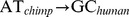 and 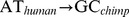 substitutions in regions identified as human hotspots. If the hotspots are completely different, we expect to see an excess of 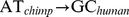 over 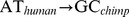 substitutions in human hotspots. Determining which species experienced the mutation, however, requires the DNA sequence of a related species as outgroup. For PAR1, however, the sequence assembly is less than 4% complete for any primate other than human and chimpanzee. Therefore, while the inability to determine the direction of the mutation reduces power to detect differences, we would still expect to observe an excess of 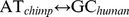 over 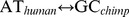 substitutions in human hotspots (if they are different from those in chimpanzee).

To quantify the relationship between substitution and recombination rate, we modelled substitution rates using a linear model with recombination rate, GC content and CpG content as explanatory variables. We performed this analysis in 2 kb intervals, the approximate size of a hotspot [80], using the HapMap2 LD-based map [29]. We considered all six mutational possibilities separately: the two types of transition (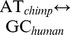 and 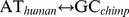) and four types of transversion (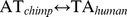, 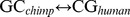, 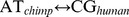, and 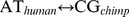). Substitution rates between the different mutational types are highly correlated with each other, and may reflect systematic differences between loci, such as variable mutation rate and chromatin context, some of which may also influence recombination rate [35], [81], [82]. To control for such systematic differences in mutation rates between loci, we modelled the substitution rate in each mutational class as the dependent variable, and included the substitution rate in all other mutational classes as explanatory variables (together with human recombination rate, GC-content and CpG content). This approach is likely to be conservative, if recombination influences both transitions and transversions towards GC bases.

Table 1 summarizes the effect size and p-value of the human recombination rate explanatory variable for each mutational class in unique DNA. Human recombination rate correlates with the rate of 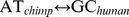 transitions, independently of the other factors we considered. This is consistent with previous studies [72], [73], and is expected based on our results above for sites segregating in human populations. Specifically, these results suggest that recombination is a driver of fixed substitutions towards GC in the PAR, even measured over millions of years, a result observed previously for the autosomes [41], [74]. A significant effect of 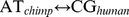 transversions was not observed. This may be because there are 2.6 fold fewer 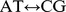 transversions, leading to lower power to detect true associations. It may also be because allowing transitions as an explanatory variation in the regression reduces our power further.

**Table 1:** Results of predicting divergence rate of different types of substitutions from human recombination rate, after regressing out effects of GC content, CpG content and the divergence rate of other types of substitutions in unique DNA.

	Type of substitution Chimpanzee	Human	Frequency per base in PAR1 (95% CI)	Effect size for human recombination rate per 10 cM/Mb (s.e.)	P-value
Transitions	AT	GC	0.37%–0.46%	0.11% (0.03%)	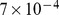
	GC	AT	0.34%–0.43%	0.03% (0.03%)	0.33
Transversions	GC	CG	0.27%–0.36%	0.03% (0.03%)	0.19
	GC	TA	0.15%–0.19%	0.01% (0.02%)	0.58
	AT	CG	0.12%–0.16%	0.00% (0.01%)	0.66
	AT	TA	0.08%–0.11%	0.00% (0.01%)	0.95

Mutations potentially due to the deamination of 5-methyl Cytosine in a CpG context in either species were excluded. Only 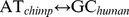 transitions are significantly correlated with human recombination rate.

However, while human recombination rate is strongly correlated with GC-biased transitions in humans, there is no evidence that it is correlated with GC-biased transitions in chimpanzee (Table 1) in the same way, because recombination does not show a symmetric association with 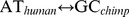 transitions. Since our results above establish that human recombination hotspots in the PAR are associated with elevation of GC substitution rates, if these sites were also hotspots in chimpanzee, we would expect to see a similar signal in that species also. Because we do not, we deduce that recombination patterns have changed strongly in the PAR between humans and chimpanzee. To investigate this further, we estimated the increase in the rate of GC-biased transitions in each species in the hottest and coldest 15% of human loci in the PAR, relative to regions with intermediate rates.

**Figure 7:**
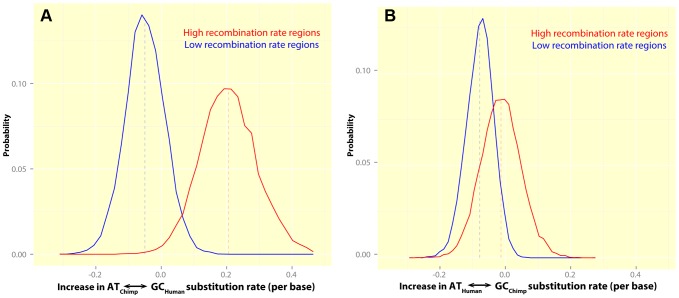
The rate of GC-biased substitutions in human and chimpanzee, in human hotspots, suggests no sharing of human and chimpanzee hotspots in PAR1. These plots show histograms for the estimated increase in the rate of GC-biased transition substitutions in regions overlapping human hotspots (red) and coldspots (blue), relative to the rest of PAR1 in: (A) Human: High recombination rate regions show a significant excess of GC-biased transitions in humans (+0.21% per base) while low recombination rate regions show a lower substitution bias towards GC transitions (−0.05% per base) relative to regions with intermediate rates. The difference between hotspots and coldspots is significant (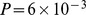). (B) Chimpanzee: There is no systematic increase in the rate of GC-biased transitions in chimpanzee (−0.01% per base) in regions containing human hotspots. The difference between hotspots and coldspots is not significant (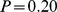).

Figure 7 shows that the hottest human regions have significantly greater accumulation of GC-biased transitions than the coldest regions (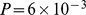), which is not the case for the chimpanzee (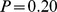). The coldest human regions have a comparably reduced rate of GC-biased transitions in both humans (−0.05% per base) and chimpanzees (−0.09% per base), suggesting that the coldest regions may be shared between the two species. This is consistent with previous work in the autosomes [41], [83], showing that certain regions (e.g. genic regions) show reduced recombination rate in both human and chimpanzee but that no shared hotspots exist. Finally, human hotspots show significantly greater rate of GC-biased transitions in human than in chimpanzee (Figure 7, 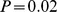). In fact, in agreement with the idea of no chimpanzee hotspot activity at human hotspots, the hottest human regions have no increase in GC-biased transitions in the chimpanzee (estimated excess in chimpanzee is −0.01% per base, relative to +0.21% per base in human). This observation that hotspots are almost certainly different in PAR1 between humans and chimpanzees is consistent with our finding that PRDM9 positions hotspots in this region.

Finally, we investigated whether hotspot heat can be predicted using the observed substitution patterns. Current approaches, such as the building of LD-based maps, require multiple individuals from a species to be genotyped or sequenced. Since such data are currently not available for the PAR in most organisms, an ability to build recombination maps using only the reference sequence of closely related species could provide a preliminary method to analyse recombination. We found that the ‘optimal’ linear model using the human-chimpanzee divergence patterns (Materials and Methods) explains 23% of the variance in the LD-based map (Table S5). While the variance explained may seem low at first, it is, in fact, in line with expectations. This is because LD-based maps capture recombination in the last thousands of generations [84] while the rate predicted from substitution patterns averages recombination since the human-chimpanzee split. If hotspots are turning over at the same rate in the PAR as in the autosomes, they are being replaced every 1 to 2 million years [54]. Given a human-chimpanzee speciation time between 5.5 and 7 million years ago [85], the LD-based maps are expected to comprise only about a third to a seventh of the recombination reflected in the substitution-based approach. We found that diversity data can also be used to estimate a genetic map, albeit at a broader scale (Figure S7).

## Discussion

In this work, we have built the most fine-scale genetic map to date from directly inferred crossovers for the human PAR1. We used this map to validate, for the first time, the previously built LD-based genetic map in this region, which localises recombination to a resolution close to the size of a hotspot. We also show the existence of biological differences between LD-based maps in different populations. We hope that these resources will promote research in this gene-rich and fast-evolving region, which currently remains under-represented in both linkage studies and on genotyping chips used in large-scale disease association scans.

Our analysis indicates that, in contrast with evidence currently available for the mouse [46], PRDM9 indeed plays a powerful role in positioning recombination events in the human PAR1. PRDM9 binding sites, and target motifs, mark crossover hotspots. In turn, these hotspots are sites of very rapid – much more rapid than on the autosomes – evolution of base content towards becoming more GC rich. Thus, as has been seen in other species [77], recombination is a rapid and powerful driver of sequence evolution in the PAR. Moreover, by using GC change as a marker of recombination sites, we observe indirectly that chimpanzee hotspots and human hotspots must show little or no overlap in PAR1, without being able to directly identify such hotspot positions in chimpanzee. This signal cannot be due to recombination only in female meiosis, because our PAR1 maps are dominated (90%) by male recombination. Moreover, the exceptionally rapid sequence evolution we see in PAR1 implies evolution driven by male meiosis, because recombination in female meioses does not occur at an unusually high rate in this region.

In many ways, PAR1 has a recombination profile in male meiosis resembling a miniature autosome, with an elevated crossover rate near the telomere. However, we observe a key difference in that a relatively high rate appears maintained throughout most of the region, without recombination coldspots as seen in the autosomes. A clue to what might be going on is perhaps given by the examination of mutations near the positions of the 13-bp motif CCnCCnTnnCCnC in PAR1, which revealed extreme skews in frequency spectra with almost no high frequency mutations toward AT bases and a U-shaped distribution of mutations towards GC bases, particularly for mutations within 25 bp of the motif (Figure 6). Recombination is the only known force able to produce such a strong skew, and our analysis shows that an order of magnitude higher fraction of these motifs form hotspots in PAR1, relative to the autosomes. This hypothesis has implications for how PAR1 manages to maintain such a uniquely high crossover rate. Firstly, it may imply a chromatin configuration in meiosis that facilitates access by PRDM9 to a high fraction of its binding sites. For instance, mouse chromatin axes are physically longer in PAR1 than the autosomes, also by an order of magnitude, potentially enabling greater access to recombination-initiating proteins [42]. Secondly, it would imply that a high fraction of bound sites go on to become recombination-promoting loci. Thus, we suggest that in humans, PRDM9 remains responsible for positioning recombination events, but that other factors may aid this protein in producing a high overall crossover rate.

We note that it is not clear our results are in contradiction with the finding of *Prdm9*-independent hotspots in the mouse PAR. For example, it may be that a back-up mechanism, independent of PRDM9, exists to ensure crossover occurs in the PAR. This back-up mechanism might, speculatively, be identical in the two mammals, but play a much larger role in mouse meiosis than in humans. This seems plausible to us based on PRDM9 binding target characteristics in the two species – the human PRDM9 target is GC-rich [54], like the PAR, and accordingly the PAR has many PRDM9 binding motifs. In contrast, studied mouse *Prdm9* alleles recognize much more AT-rich motifs [46]. There were no matches, for instance, to the mouse motif TCnTGnTnCTT [86] in the section of mouse PAR assembled so far (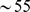 kb), whereas there were 9 matches to the human motif CCnCCnTnnCCnC, which has the same number of specified bases. The mouse motif is thus potentially rare or absent in its PAR, and likely to become rapidly eroded due to the phenomenon of gcBGC we have discussed here. Recombination in humans has been shown to lead to loss of PRDM9-binding motifs that become hotspots, via biased gene conversion (with a mechanism distinct from that of gcBGC). This phenomenon has been proposed to place evolutionary pressure on PRDM9 to evolve rapidly, as it is observed to do [52], to avoid eventual depletion of crossover locations essential for meiosis. The PAR represents an obvious genomic location where this problem might be especially acute, due to its small size and high recombination rate, perhaps even contributing to the rapid evolution of PRDM9. However, whether such rapid loss is occurring in the PAR in humans has not been possible for us to test, due to lack of statistical power. Interestingly, the force of gcBGC could even oppose the loss of PRDM9 target motifs, by creating other motifs, because human PRDM9 binding target motifs are GC-rich. Similarly, minisatellite mutation mechanisms may expand the number of PRDM9 binding sites in PAR1, by duplicating motif copies [58]–[60]. It is not clear, however, if these mechanisms can dominate over motif loss, and more study is required to better understand the evolutionary properties of PRDM9 binding sites, and more generally the DNA sequence, through time, in this intriguing region.

## Materials and Methods

### Building a pedigree-based map for PAR1

We have used genotype data from 135 previously published African-American pedigrees [47]. The pedigrees were drawn from cohorts in the CARe consortium: 70 families from the Jackson Heart Study (JHS) and 65 families from the Cleveland Family Study (CFS). After quality control filtering, 209 markers were available for CFS samples and either 215 or 180 or 192 markers for different subsets of JHS samples (more details are provided in Text S1). A union of these SNPs was performed, resulting in 220 SNPs, which were used to build the map in PAR1. A listing of these SNPs is provided in Dataset S1.

Each family had at least two children, and at least one parent genotyped. Crossovers were identified using an adaptation of the Lander-Green algorithm [87] that accommodates genotyping error and significant degrees of missing data, and has been published previously [47]. The algorithm has been summarized in Text S1 for completeness.

To increase power to detect crossovers near the pseudoautosomal boundary, we have included 100 SNPs from the X chromosome (Text S1). Fathers and sons were modelled to have one X-specific chromosome proximal to the pseudoautosomal boundary, and one ‘dummy’ chromosome with a fixed genotype sequence and no recombination. This improves the detection of both paternal and maternal crossovers near the pseudoautosomal boundary.

The algorithm estimates the posterior probability of crossover in each SNP interval across all parents. To build a male map, we add the probability of crossover for each SNP interval for all fathers, and divide by the total number of male meioses. We repeat this process for mothers to produce a female map. We post-process the cumulative posterior probability distribution of crossover over all SNP intervals for each parent to identify individual crossovers (Text S1).

The male and female genetic maps are provided in Dataset S2. The crossovers where both endpoints mapped into our regions of marker coverage are provided in Dataset S3 (male) and Dataset S4 (female).

### HapMap2 LD-based maps for PAR1

The HapMap2 population-averaged LD-based map for PAR1 was downloaded from:

https://mathgen.stats.ox.ac.uk/impute/impute_v1.html#Download

Population-specific recombination maps were kindly provided by Colin Freeman from the Wellcome Trust Centre for Human Genetics, Oxford University.

LiftOver tool [88] was used to convert maps in builds 35/36 to builds 36/37.

### Measuring PRDM9 binding in PAR1 in human cells

A cDNA for the human *PRDM9* B-allele was synthesised and cloned into a transient expression vector (pLEXm [89]) with an N-terminal Venus YFP tag. Large-scale transfections were performed in HEK293T cells as described [89]. Cells were harvested 72 hours after transfection and processed for ChIP-seq according to an online protocol used for the ENCODE project by the laboratory of Rick Myers [90]. Immunoprecipitation was performed using an Abcam rabbit polyclonal ChIP-grade anti-GFP antibody (ab290), and two technical replicates were performed. Uncrosslinked total chromatin DNA (without immunoprecipitation) was sequenced as a control sample. ChIP-DNA and control DNA were sequenced using 180 million paired 51 bp Illumina reads per replicate. Reads were aligned to hg19 and PCR duplicates were removed. Peak calling was performed using an in-house, maximum-likelihood-based peak calling algorithm that uses fragment coverage information from both sequencing replicates and the total chromatin control. Peaks were called at a p-value cutoff of 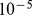. Further details of the protocol are provided in Text S1. The peaks are listed in Dataset S5. A separate manuscript describing the ChIP-seq results for the rest of the genome is in preparation.

### Detection of substitutions between humans and chimpanzee

To detect substitutions on the human and chimpanzee lineages, we downloaded the GRCh37-CHIMP2.1.4 (release 70) alignment available from Ensembl. The alignment was restricted to regions with accurate expected LD-based map rates (we removed the first and last 50 markers in the HapMap2 LD-based map, out of a total of 1385 markers, since power is reduced to detect the breakdown of LD there.). After this, the alignment contains approximately 1.2 Mb of sequence. For this analysis, we divided PAR1 into 2 kb regions, and included only those regions for analysis where at least 1 kb of the sequence was not repeat-masked and aligned without deletions or missing data on either lineage. A small number of regions were observed with total human/chimpanzee divergence greater than 5% and up to 11%. They were strongly clustered and represented clear outliers in the divergence distribution. These were filtered out from the analysis as they are not representative of PAR1 in general, and because we suspect that they represent mismapped or misaligned regions.

### Linear model for recombination rate prediction

A stepwise search was performed to predict recombination rate using a linear model. The Aikake Information Criterion (AIC) was used to perform model selection and minimize overfitting. The full set of explanatory variables considered were the GC-content fraction, CpG content fraction and divergence rates for each of 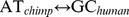, 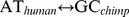, 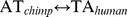, 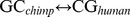, 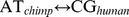, and 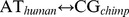 substitutions. Models were fit for substitutions in non-repeat DNA only.

### Ethics statement

Informed consent was provided by all the individuals participating in the study, and was approved by all of the institutions responsible for sample collection.

## Supporting Information

Figure S1 Broad-scale pedigree-based maps for PAR1. Sex-specific pedigree-based genetic maps smoothed to 250 kb to reveal broad-scale trends. The male map (blue) shows a decreasing overall trend away from the telomere, while the female map (red) shows an increase away from the telomere. The male map also shows a modest increase in rates close to the pseudoautosomal boundary. Vertical black tick marks show marker positions. Repeat content is calculated at a 10 kb scale. Physical coordinates are in build 36.
(PDF)

Figure S2 Recombination rate in PAR1 near copies of CCnCCnTnnCCnC in L2 elements. The presence of the canonical human 13-bp motif CCnCCnTnnCCnC predicts a strong local increase in recombination rate in the LD-based map. This plot shows rates around the 4 instances of L2 elements containing an exact match to the motif and where rates could be estimated (plotted in 2 kb intervals, and a 100 bp moving window).
(PDF)

Figure S3 Recombination rate in PAR1 around PRDM9 binding sites identified by ChIP-seq. Rate in the HapMap2 population-averaged LD-based map in the vicinity of ChIP-seq binding locations of the PRDM9 reference allele (B allele) in PAR1 for: (a) binding locations containing at least one close match to the PRDM9 binding motif (b) binding locations without a close match to the motif.
(PDF)

Figure S4 The frequency spectra of derived alleles in Chr 20. The frequency spectra of derived alleles in Chr 20 shows a U-shaped distribution, and an excess of high frequency GC-increasing mutations, relative to GC-reducing and GC-neutral mutations. However, the differences between the mutations are much greater in PAR1 (Figure 5a).
(PDF)

Figure S5Comparison of the allele frequency distributions of AT
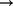
GC and GC
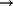
AT mutations in two regions of PAR1. The most telomeric region (200 kb–700 kb) has a high male rate (24 cM/Mb), and is very cold in the female map (0.5 cM/Mb), with a sex-averaged rate of about 12 cM/Mb. The region closest to the pseudoautosomal boundary (2.2 Mb–2.7 Mb) is moderately hot in both males and females (10 cM/Mb and 5 cM/Mb), with a significantly lower sex-averaged rate of about 7.5 cM/Mb. The significantly stronger bias towards higher AT
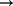
GC allele frequencies in the telomeric region (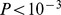) shows that (a) Hotter regions in PAR1 are subject to greater GC-bias, confirming a quantitative association between recombination rate and gcBGC in the PAR, and (b) Male recombination is the dominant force leading to gcBGC in PAR1, and that the patterns of gcBGC cannot be explained by female recombination alone.(PDF)

Figure S6Comparison of the allele frequency distributions of AT
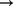
GC and GC
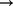
AT mutations within 50 bp of the motif CCnCCnTnnCCnC in two regions of PAR1. Comparison of the allele frequency distributions of AT
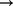
GC and GC
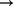
AT mutations *within 50 bp of the motif CCnCCnTnnCCnC* in two 500 kb regions of the PAR, relative to those mutations throughout the respective regions (including both transitions and transversions). Recombinogenic activity of the motif is at least as high in the more+ telomeric region of PAR1 as it is in the region closest to the pseudoautosomal boundary.(PDF)

Figure S7PAR1 genetic map estimated using the allele frequency spectra of derived alleles in human populations. A map estimated using a linear model based on 70th percentile of the derived allele frequency of AT
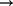
GC transitions and transversions in 1000 Genomes relative to the sex-average pedigree-based map in African-Americans (smoothed at 250 kb scale with a 10 kb moving window).(PDF)

Table S1 Resolution of crossovers identified using African-American pedigrees.
(PDF)

Table S2 Male and female broad-scale rates in PAR1.
(PDF)

Table S3 Differences in male broad-scale rates in PAR1.
(PDF)

Table S4 Differences in female broad-scale rates in PAR1.
(PDF)

Table S5 Linear model for predicting recombination rate from sequence features.
(PDF)

Dataset S1 Markers in the pedigree-based map. Columns 1 and 2 are the rsID and Build 36 positions of the SNPs respectively. Columns 3–6 show whether the SNPs were included in each of the constituent studies, with “1” representing inclusion, and “0” not.
(TXT)

Dataset S2 Male and female pedigree-based maps. Column 1 is the Build 36 position, and columns 2 and 3 are the cumulative genetic distance in Morgans up to the marker in column 1.
(TXT)

Dataset S3 Paternal Crossover locations. Columns 1 and 2 are the start and end points of paternal crossovers in build 36.
(TXT)

Dataset S4 Maternal Crossover locations. Columns 1 and 2 are the start and end points of maternal crossovers in build 36.
(TXT)

Dataset S5 PRDM9 binding sites. Binding sites inferred by a ChIP-seq protocol in human cells. Columns 1 and 2 are the start and end points of inferred binding peaks in Build 37. Column 3 is the p-value of the peak call.
(TXT)

Text S1 Supplementary text. Details of pedigree map-building work, and the ChIP-seq experimental protocol to measure PRDM9 binding in human cells.
(PDF)
