# Supplementary figures and images for: Recombination in the Human Pseudoautosomal Region PAR1
Source: PLoS Genet. 2014 Jul 17;10(7):e1004503. doi: 10.1371/journal.pgen.1004503 (PMC4102438; doi:10.1371/journal.pgen.1004503)

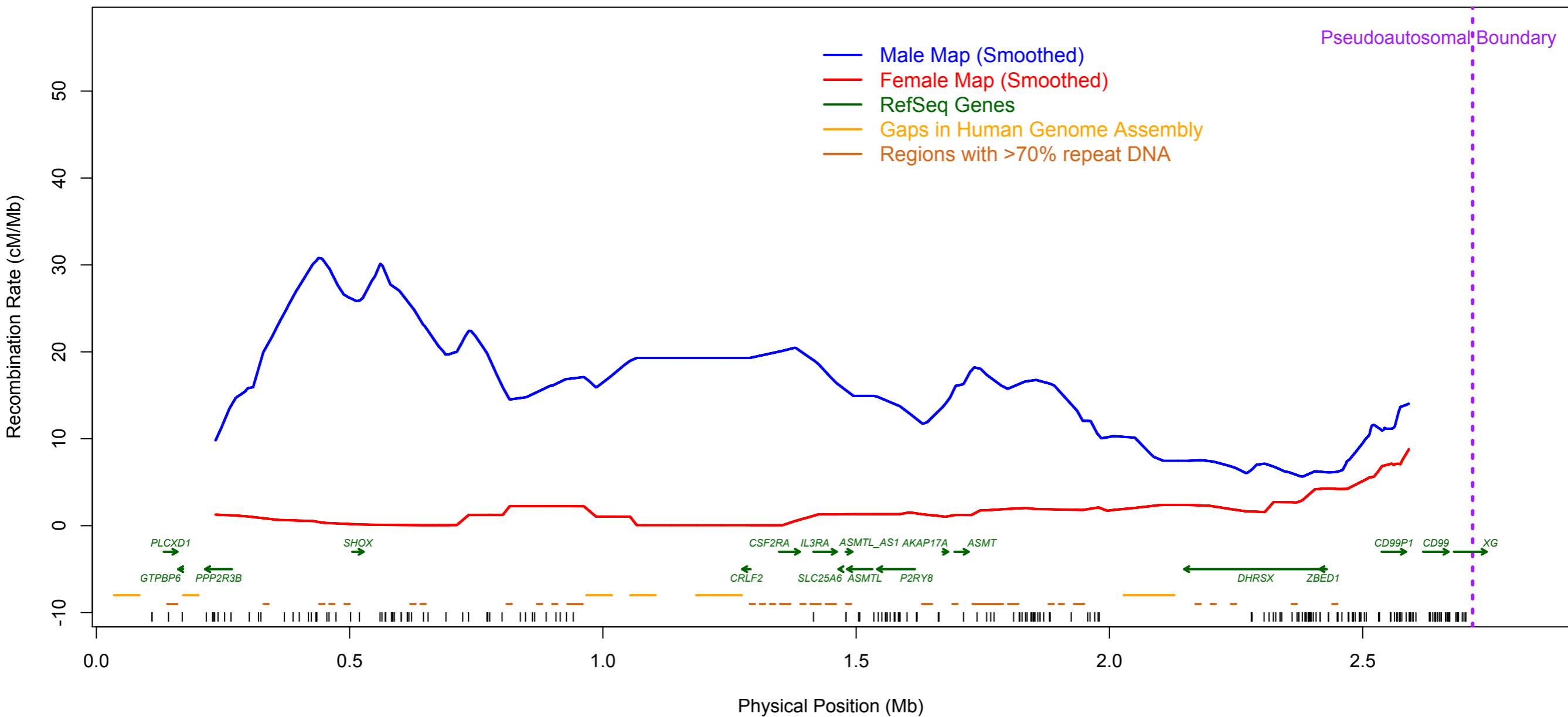

Supplement: Figure S1 — Broad-scale pedigree-based maps for PAR1. Sex-specific pedigree-based genetic maps smoothed to 250 kb to reveal broad-scale trends. The male map (blue) shows a decreasing overall trend away from the telomere, while the female map (red) shows an increase away from the telomere. The male map also shows a modest increase in rates close to the pseudoautosomal boundary. Vertical black tick marks show marker positions. Repeat content is calculated at a 10 kb scale. Physical coordinates are in build 36. (PDF) [file pgen.1004503.s001.pdf]

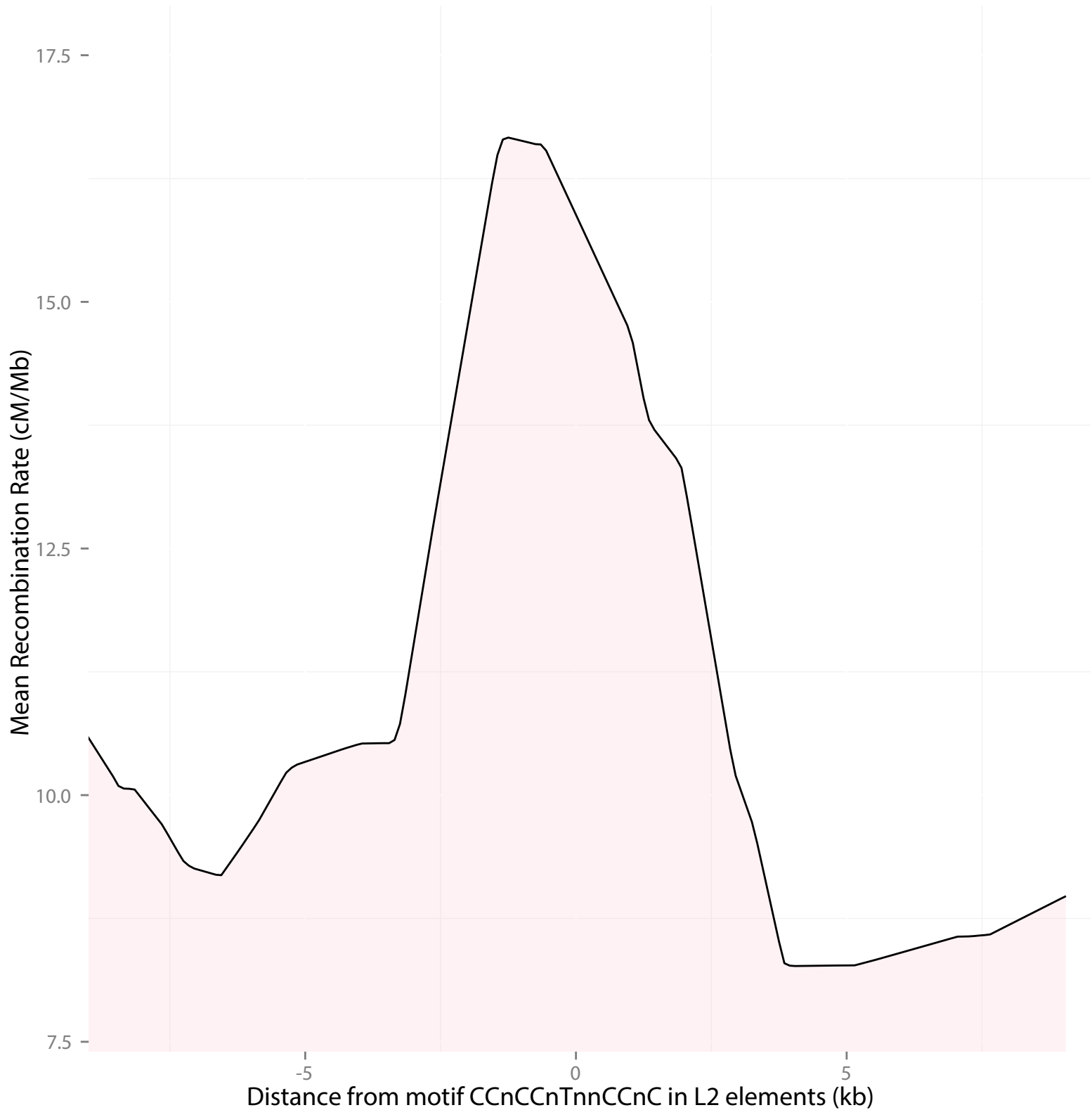

Supplement: Figure S2 — Recombination rate in PAR1 near copies of CCnCCnTnnCCnC in L2 elements. The presence of the canonical human 13-bp motif CCnCCnTnnCCnC predicts a strong local increase in recombination rate in the LD-based map. This plot shows rates around the 4 instances of L2 elements containing an exact match to the motif and where rates could be estimated (plotted in 2 kb intervals, and a 100 bp moving window). (PDF) [file pgen.1004503.s002.pdf]

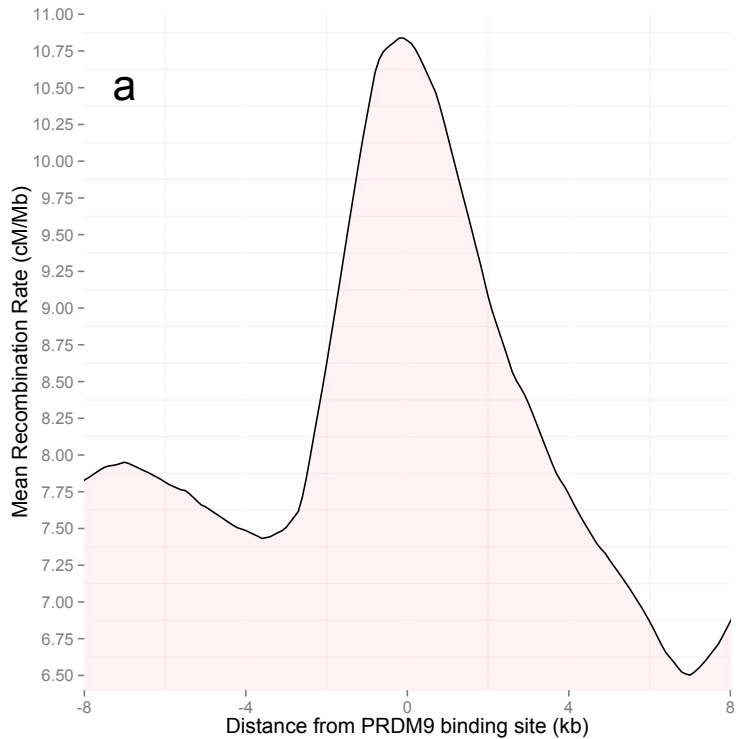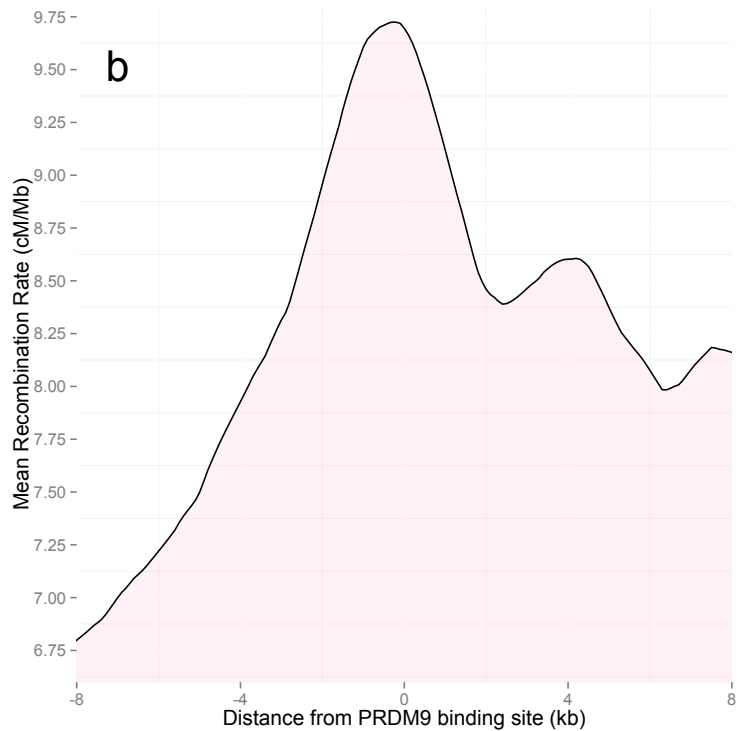

Supplement: Figure S3 — Recombination rate in PAR1 around PRDM9 binding sites identified by ChIP-seq. Rate in the HapMap2 population-averaged LD-based map in the vicinity of ChIP-seq binding locations of the PRDM9 reference allele (B allele) in PAR1 for: (a) binding locations containing at least one close match to the PRDM9 binding motif (b) binding locations without a close match to the motif. (PDF) [file pgen.1004503.s003.pdf]

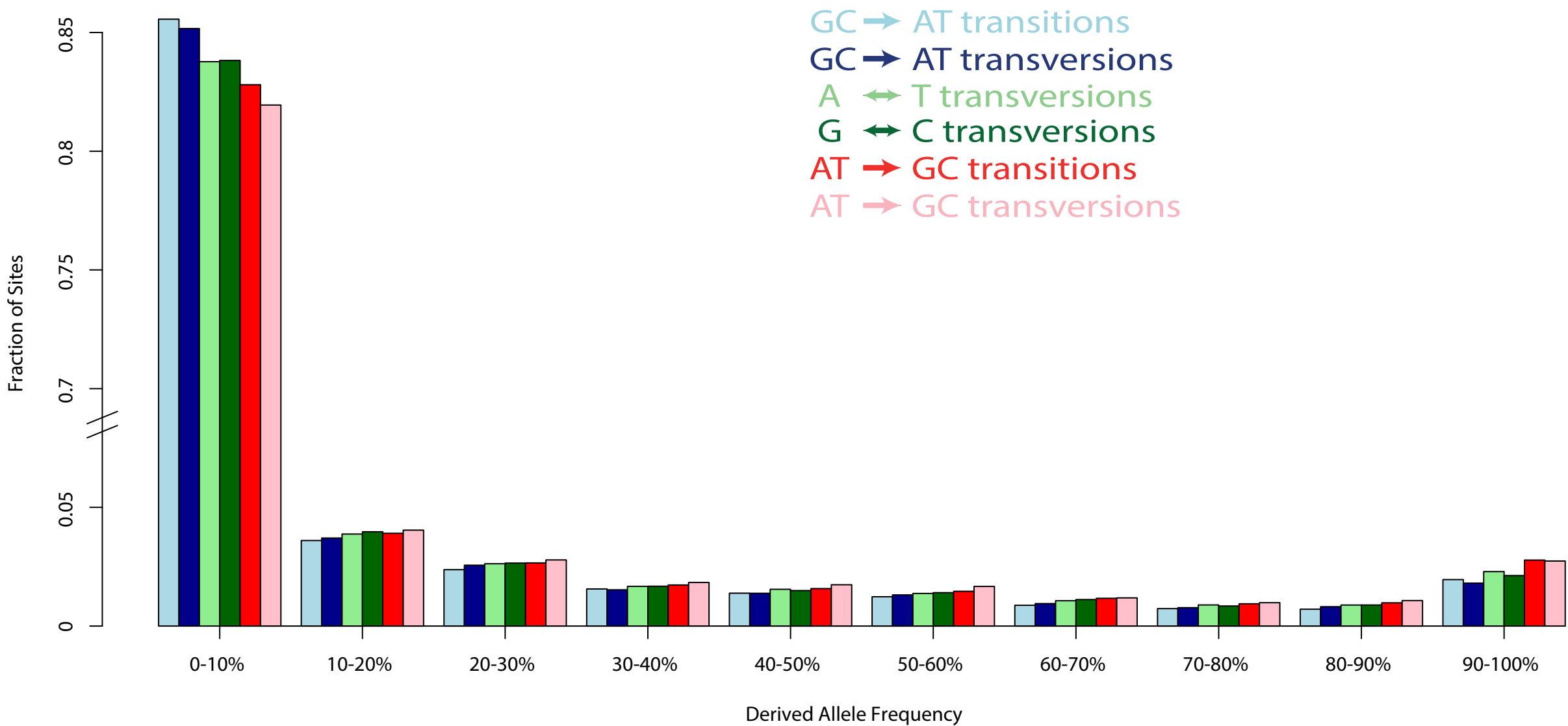

Supplement: Figure S4 — The frequency spectra of derived alleles in Chr 20. The frequency spectra of derived alleles in Chr 20 shows a U-shaped distribution, and an excess of high frequency GC-increasing mutations, relative to GC-reducing and GC-neutral mutations. However, the differences between the mutations are much greater in PAR1 (Figure 5a). (PDF) [file pgen.1004503.s004.pdf]

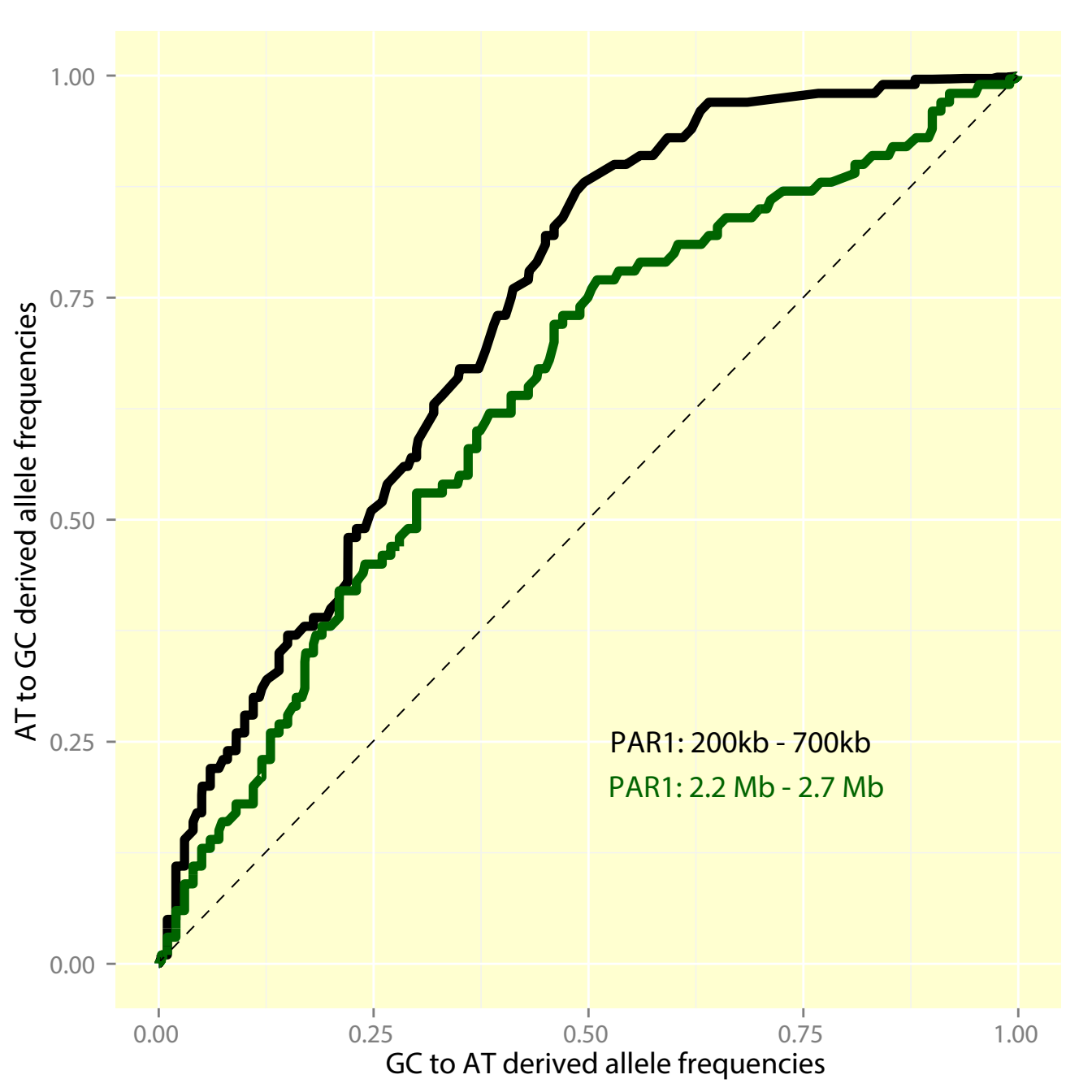

Supplement: Figure S5 — Comparison of the allele frequency distributions of ATGC and GCAT mutations in two regions of PAR1. The most telomeric region (200 kb–700 kb) has a high male rate (24 cM/Mb), and is very cold in the female map (0.5 cM/Mb), with a sex-averaged rate of about 12 cM/Mb. The region closest to the pseudoautosomal boundary (2.2 Mb–2.7 Mb) is moderately hot in both males and females (10 cM/Mb and 5 cM/Mb), with a significantly lower sex-averaged rate of about 7.5 cM/Mb. The significantly stronger bias towards higher ATGC allele frequencies in the telomeric region () shows that (a) Hotter regions in PAR1 are subject to greater GC-bias, confirming a quantitative association between recombination rate and gcBGC in the PAR, and (b) Male recombination is the dominant force leading to gcBGC in PAR1, and that the patterns of gcBGC cannot be explained by female recombination alone. (PDF) [file pgen.1004503.s005.pdf]

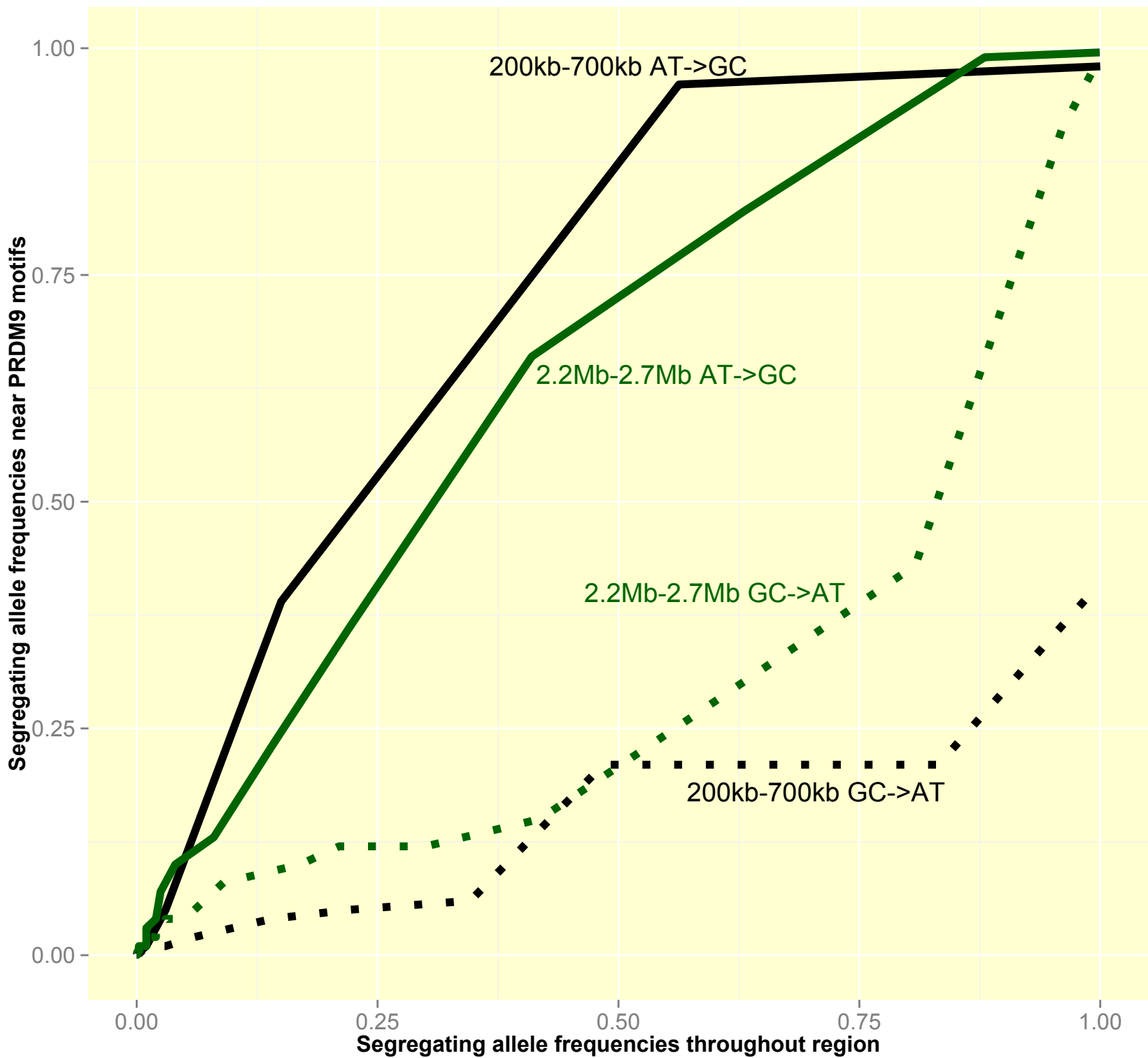

Supplement: Figure S6 — Comparison of the allele frequency distributions of ATGC and GCAT mutations within 50 bp of the motif CCnCCnTnnCCnC in two regions of PAR1. Comparison of the allele frequency distributions of ATGC and GCAT mutations within 50 bp of the motif CCnCCnTnnCCnC in two 500 kb regions of the PAR, relative to those mutations throughout the respective regions (including both transitions and transversions). Recombinogenic activity of the motif is at least as high in the more+ telomeric region of PAR1 as it is in the region closest to the pseudoautosomal boundary. (PDF) [file pgen.1004503.s006.pdf]

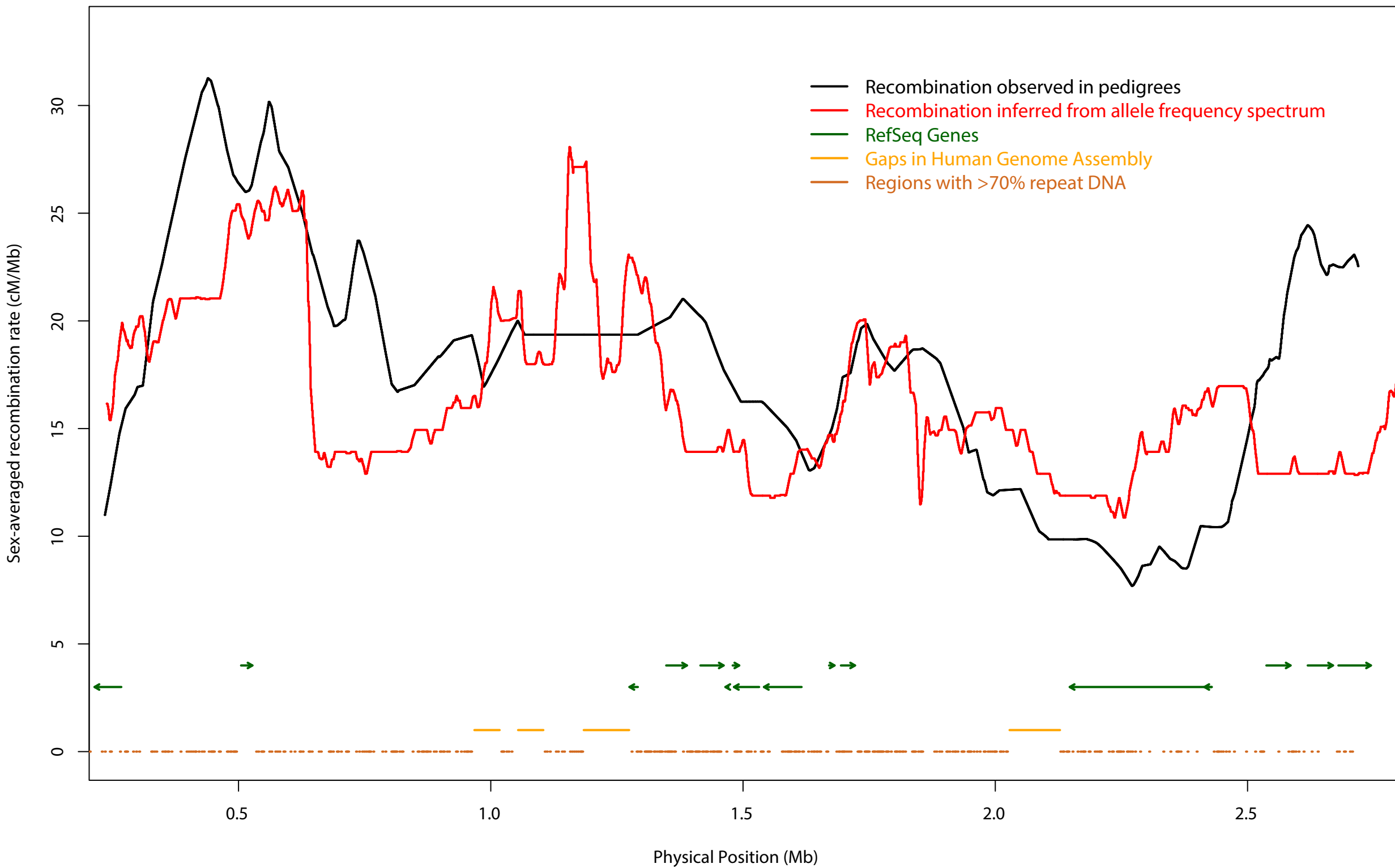

Supplement: Figure S7 — PAR1 genetic map estimated using the allele frequency spectra of derived alleles in human populations. A map estimated using a linear model based on 70th percentile of the derived allele frequency of ATGC transitions and transversions in 1000 Genomes relative to the sex-average pedigree-based map in African-Americans (smoothed at 250 kb scale with a 10 kb moving window). (PDF) [file pgen.1004503.s007.pdf]
