# Supplementary material for: Recombination in the Human Pseudoautosomal Region PAR1
Source: PLoS Genet. 2014 Jul 17;10(7):e1004503. doi: 10.1371/journal.pgen.1004503 (PMC4102438; doi:10.1371/journal.pgen.1004503)
Supplement: Table S1 — Resolution of crossovers identified using African-American pedigrees. (PDF) [file pgen.1004503.s008.pdf]

Resolution	#Paternal Events	#Maternal Events
< 10 kb	0	0
$10 - 20$ kb	3	1
$20 - 50$ kb	15	5
$50 - 100$ kb	23	4
$100 - 200$ kb	27	3
$200 - 500$ kb	23	3
> 500 kb	35	1
Total	126	17

Table S1: Resolution of paternal and maternal crossovers that have both end-points mapping into our region of marker coverage
