# Supplementary material for: Recombination in the Human Pseudoautosomal Region PAR1
Source: PLoS Genet. 2014 Jul 17;10(7):e1004503. doi: 10.1371/journal.pgen.1004503 (PMC4102438; doi:10.1371/journal.pgen.1004503)
Supplement: Table S2 — Male and female broad-scale rates in PAR1. (PDF) [file pgen.1004503.s009.pdf]

	Male (cM)	Female (cM)
200 kb – 700 kb	11.9	0.25
700 kb – 1.2 Mb	8.5	0.58
1.2 Mb – 1.7 Mb	8.4	0.35
1.7 Mb – 2.2 Mb	6.2	1.1
2.2 Mb – 2.7 Mb	5.1	2.4

Table S2: Male and female broad-scale rates in PAR1. The sub-telomeric 200 kb were removed due to low power to identify crossovers.
