# Supplementary material for: Recombination in the Human Pseudoautosomal Region PAR1
Source: PLoS Genet. 2014 Jul 17;10(7):e1004503. doi: 10.1371/journal.pgen.1004503 (PMC4102438; doi:10.1371/journal.pgen.1004503)
Supplement: Table S3 — Differences in male broad-scale rates in PAR1. (PDF) [file pgen.1004503.s010.pdf]

	200 kb – 700 kb	700 kb – 1.2 Mb	1.2 Mb – 1.7 Mb	1.7 Mb – 2.2 Mb	2.2 Mb – 2.7 Mb
200 kb – 700 kb	11.9 cM	0.039	0.033	1.8×10^{-3}	4.0×10^{-4}
700 kb – 1.2 Mb		8.5 cM	0.48	0.059	0.021
1.2 Mb – 1.7 Mb			8.4 cM	0.07	0.029
1.7 Mb – 2.2 Mb				6.2 cM	0.25
2.2 Mb – 2.7 Mb					5.1 cM

Table S3: This table shows the evidence for a *decrease in rate away from the telomere* in the *male* PAR1. The diagonal elements report the estimated rate in each region. The other cells report the p-value of testing the null hypothesis $r_i = r_j$, against $r_i > r_j$ for row i and column j, where r_i and r_j are the broad-scale rates in the corresponding regions. The p-values were estimated using 5000 bootstrap samples over the set of fathers included in the map.
