# Supplementary material for: Recombination in the Human Pseudoautosomal Region PAR1
Source: PLoS Genet. 2014 Jul 17;10(7):e1004503. doi: 10.1371/journal.pgen.1004503 (PMC4102438; doi:10.1371/journal.pgen.1004503)
Supplement: Table S4 — Differences in female broad-scale rates in PAR1. (PDF) [file pgen.1004503.s011.pdf]

	200 kb – 700 kb	700 kb – 1.2 Mb	1.2 Mb – 1.7 Mb	1.7 Mb – 2.2 Mb	2.2 Mb – 2.7 Mb
200 kb – 700 kb	0.25 cM	0.22	0.42	0.03	4.0×10^{-4}
700 kb – 1.2 Mb		0.58 cM	0.61	0.22	0.01
1.2 Mb – 1.7 Mb			0.35 cM	0.10	0.003
1.7 Mb – 2.2 Mb				1.1 cM	0.05
2.2 Mb – 2.7 Mb					2.4 cM

Table S4: This table shows the evidence for an *increase in rate near the pseudoautosomal boundary* in the *female* PAR1. The diagonal elements report the estimated rate in each region. The other cells report the p-value of testing the null hypothesis $r_i = r_j$, against $r_i < r_j$ for row i and column j, where r_i and r_j are the broad-scale rates the corresponding regions. The p-values were estimated using 5000 bootstrap samples over the set of mothers included in the map.
