# Supplementary material for: Recombination in the Human Pseudoautosomal Region PAR1
Source: PLoS Genet. 2014 Jul 17;10(7):e1004503. doi: 10.1371/journal.pgen.1004503 (PMC4102438; doi:10.1371/journal.pgen.1004503)
Supplement: Table S5 — Linear model for predicting recombination rate from sequence features. (PDF) [file pgen.1004503.s012.pdf]

Type of substitution Chimpanzee	Human	Effect Size (Non-repeat DNA)	P-value (Non-repeat DNA)
AT	GC	8.5	1×10^{-4}
GC	CG	6.1	0.02
GC content fraction		-15.5	0.08
CpG content fraction		39.1	0.12
Intercept		8.5	0.02

Table S5: Genetic maps can be built using substitution patterns between closely related species: this table reports the optimal linear model for predicting hotspot heat in cM/Mb while preventing over-fitting of data (using the AIC criterion for model selection) for predicting hotspot heat. The regressors allowed in the step-wise search were GC-content fraction, CpG content fraction and the divergence rate for each category of substitution. The R^2 of this model is 23% in unique DNA.
