# Supplementary material for: Recombination in the Human Pseudoautosomal Region PAR1
Source: PLoS Genet. 2014 Jul 17;10(7):e1004503. doi: 10.1371/journal.pgen.1004503 (PMC4102438; doi:10.1371/journal.pgen.1004503)
Supplement: Text S1 — Supplementary text. Details of pedigree map-building work, and the ChIP-seq experimental protocol to measure PRDM9 binding in human cells. (PDF) [file pgen.1004503.s018.pdf]

Recombination in the human pseudoautosomal region PAR1
Text S1

Anjali G Hinch, Nicolas Altemose, Nudrat Noor, Peter Donnelly, Simon R Myers

Contents

1 Crossover events in PAR1 in African-American families 1
1.1 Data 1
1.2 Inference of recombination in families with missing data and genotyping error 2

2 Measuring PRDM9 binding in PAR1 in a human cell line 3
2.1 Generating a YFP-PRDM9 construct 3
2.2 Transfection 4
2.3 ChIP-seq 4
2.4 Sequence filtering and processing 5
2.5 Calling PRDM9 binding peaks 6

1 Crossover events in PAR1 in African-American families

1.1 Data

In this study we analyzed a total of 135 nuclear families, representing 672 meioses. The studies that contributed samples were cohorts comprising the CARe consortium: the Jackson Heart Study (JHS) and the Cleveland Family Study (CFS). 70 families were drawn from the JHS study, and 65 families from the CFS study. Further genotyping was performed for 58 JHS samples [1], which were members of 17 previously included JHS families. All samples were genotyped on the Affymetrix 6.0 array. Data curation was done independently for each study. After filtering, we had data in PAR1 for 215 SNPs for the original JHS samples, 180 SNPs for a subset of JHS family members who were genotyped as part of the Atherosclerosis Risk in Communities study, 209 SNPs for CFS and 192 SNPs for the additional JHS samples. We built the pedigree based map with the union of these SNPs, comprising a total of 220 SNPs.

Parents who were not genotyped were treated as missing data. SNPs not available in a cohort were also considered missing data.

The full list of SNPs is provided in an Dataset S1. Columns 1 and 2 are the rsID and Build 36 positions of the SNPs respectively. Columns 3-6 show whether the SNPs were included in each of JHS, ARIC, CFS, and the additional JHS samples, with "1" representing inclusion, and "0" not.

1.2 Inference of recombination in families with missing data and genotyping error

We have previously published this algorithm, which is an adaptation of the Lander-Green algorithm [2], and used it for inference of crossovers in African-American families [1]. We summarise the algorithm here for completeness.

Consider a trio of father, mother and child. The child inherits one chromosome from the mother and one from the father. The maternally inherited chromosome may be a mosaic of the two maternal chromosomes due to crossing over. It is completely specified by the knowledge of which of those two chromosomes was copied at each locus, and this information is called the maternal *inheritance vector* of the child. The child similarly has a paternal inheritance vector specifying which of the two paternal chromosomes was copied at each locus.

Going along a chromosome, the maternal inheritance vector flips at locus j when there is a crossover in the mother between loci $j-1$ and j. Under the assumption of no interference, the probability of crossover between loci $j-1$ and j is independent of crossover in any other locus, and the inheritance vectors are therefore first-order Markov processes. The trio can therefore be modelled as a Hidden Markov Model (HMM) with the inheritance vectors as the hidden states. Each meiosis is independent, and therefore, additional children in the family will have independent inheritance vectors.

The parental haplotypes are not known, only the genotype is known. The haplotypes are therefore also modelled as hidden states. The parental haplotypes will not, in general, be Markov processes due to linkage between loci. For most of the genome, markers have to be thinned down in order for this assumption to hold approximately. In the case of PAR1, however, the breakdown of linkage disequilibrium (LD) is rapid, and the available SNP density is relatively low, and no further thinning of markers is necessary.

We run the forward-backward algorithm [3] and use the forward and backward probabilities to calculate the posterior probability of parental haplotypes and inheritance vectors at each locus. The inferred probability of crossover between loci $j-1$ and j is simply the posterior joint probability of all states at loci $j-1$ and j that involve a change in inheritance vectors between those loci (Please see [1, 4] for the detailed calculations).

If the genetic data of one parent is unavailable, the HMM treats it as missing data, and jointly infers the parental genotype together with the inheritance vector of each child. How well the parental genotype can be imputed in this procedure depends on the number of genotyped children. In a family with 2 children we expect to see both alleles from the

missing parent half the time. That fraction is three-quarters in a family with 3 children, seven-eighths with 4 children and so on.

Due to insufficient information near the telomeres, we lose ability to detect crossovers in approximately 250 kb on each end of the chromosome under consideration. There is no escape from this issue in the sub-telomeric region of any chromosome, in general. However, as the pseudoautosomal boundary (PAB) is not actually at the end of a chromosome, the problem can be ameliorated at the PAB-end of PAR1. We do this by including genotype data from 100 SNPs from the X-specific region adjacent to the PAB. The transition matrices of mothers and daughters are unchanged. Fathers and sons are modelled to have two PAR1 haplotypes, as usual. In the X-specific region, however, they are modelled to have one X chromosome, and one 'dummy' chromosome with a fixed genotype sequence of zeroes (WLOG). No recombination is permitted in the transition matrix of the paternal inheritance vector in the X-specific region. The rest of the HMM calculations remain unchanged. This improves the detection of both paternal and maternal crossovers near the PAB.

Finally, to build a map, we add the posterior probability of crossover in each SNP interval for all fathers. We divide by the total number of male meioses to get an average map for males. We repeat this process for mothers to produce a female map. A total genetic distance of 136 M was inferred for fathers, equivalent to 0.4 M/meiosis. The total genetic distance in the female map was 18 M, equivalent to 0.05 M/meiosis.

We post-process the cumulative probability distribution of crossover across all SNP intervals for each parent to identify individual crossovers [1]. Specifically, we use a dynamic programming algorithm to identify the end points of crossovers. The end points are inferred such that the probability distribution function between the crossover end points is maximally steep and a crossover is contained with probability at least 95%. For complete details of the algorithm, including an illustration of the model, and examples of crossovers, please see [1, 4].

The male and female genetic maps and crossovers are provided in Datasets S2-S4.

2 Measuring PRDM9 binding in PAR1 in a human cell line

Here we describe the steps in the experimental protocol for expressing PRDM9 in a human cell line, followed by direct measurements of its DNA binding properties using ChIP-seq.

2.1 Generating a YFP-PRDM9 construct

A cDNA was custom synthesised to contain the full-length (2685bp) *PRDM9* transcript from the human reference genome (GRCh37), which is the B allele of *PRDM9*. 218 synonymous base changes were engineered in the exon containing the zinc finger domain in

order to distinguish the synthetic copy of *PRDM9* from the endogenous copy. This cDNA was cloned into the pLEXm transient expression vector [5] with a Venus (YFP) tag at its N-terminus (where it is least likely to interfere with the Zinc Finger domain). This construct was cloned, amplified, and isolated using an Qiagen EndoFree Plasmid Giga Kit to yield transfection-quality DNA, which was verified by restriction digestion and Sanger sequencing.

2.2 Transfection

HEK293T cells were chosen owing to their high transfection efficiency, rapid growth rate, and low-cost media requirements. Large-scale transfections were performed as described [5]. Cells were grown in DMEM media (10% FCS, 1X NEAA, 2mM L-Glut) in 200ml roller bottles at 37C/5% CO_2. A transfection cocktail was prepared for each bottle by adding 0.5 mg of chloroform-purified construct DNA to 50 ml of serum-free DMEM (1X NEAA, 2mM L-glut) and 1 mg polyethylenimine, followed by a 10-minute incubation then addition of 375 μg of kifunensine. After the cells reached 75% confluency, the media was removed from each roller bottle and replaced with 200ml low-serum DMEM (2% FCS, 1X NEAA, 2mM L-Glut) and 50ml transfection cocktail. Cells were then incubated for 72 hours to enable expression of the transfected construct. Expression was verified by placing a small aliquot of detached cells on a glass slide with DAPI and viewing them under a confocal fluorescence microscope at 20X magnification.

2.3 ChIP-seq

ChIP-seq was performed according to an online protocol produced by Rick Myers's laboratory [6], which was used to produce much of the ENCODE Project's ChIP-seq data, with several optimising modifications.

Crosslinking. Bottles were removed from the incubator and shaken vigorously to detach cells. Fresh formaldehyde was added to a final concentration of 0.75% and cells were incubated at room temperature for 15 minutes. The crosslinking reaction was stopped by adding glycine to a final concentration of 125mM. Cells were aliquoted to 50ml conical tubes, centrifuged (2000xg, 5 minutes), resuspended in cold 1X PBS, and centrifuged again. Pellets were snap frozen with dry ice then stored at -80C.

Lysis and Sonication. Frozen pellets were thawed and resuspended in cold Farnham Lysis Buffer (5mM PIPES pH 8.0, 85 mM KCl, 0.5% NP-40, 1 tablet Roche Complete protease inhibitor per 50ml) to a concentration of 20M cells per ml, then passed through a 22G needle 20 times to further lyse and homogenise them. Lysates were centrifuged and resuspended in 300 ul cold RIPA lysis buffer (1X PBS, 1% NP-40, 0.5% sodium deoxycholate, 0.1% SDS, 1 tablet Roche Complete protease inhibitor per 50ml) per 20M cells to lyse nuclei. 300ul samples were sonicated in a Bioruptor Twin sonication bath in 1.5ml eppendorf tubes at 4C for two 10-minute periods of 30 seconds on, 30 seconds off at high power. Cell debris was

removed by centrifugation (14000 rpm, 15 minutes, 4C), and supernatants were isolated and brought to a final volume of 1ml with RIPA. These chromatin preps were snap-frozen in dry ice then stored at -80C.

Immunoprecipitation. Magnetic beads were washed by adding 200ul Invitrogen Sheep Anti-Rabbit Dynabeads per sample to 800 ul cold PBS/BSA (1X PBS, 5mg/ml BSA, 1 tablet Roche Complete protease inhibitor per 50ml, filtered with 0.45 micron filter). Solutions were placed on a magnetic rack and resuspended in 1ml PBS/BSA 4 times. 5 ul Abcam rabbit polyclonal ChIP-grade anti-GFP antibody (ab290) was added and solutions were incubated overnight at 4C on a rotator. Antibody-coupled beads were washed 3 times with cold PBS/BSA and resuspended in 100ul PBS/BSA, then added to 1ml chromatin preps thawed on ice. One tube was prepared in parallel without adding beads, to yield a genomic background control sample from total chromatin. Tubes were incubated for 12 hours on a rotator at 4C, then washed 5 times for 3 minutes each with cold LiCl Wash Buffer (100mM Tris pH 7.5, 500mM LiCl, 1% NP-40, 1% sodium deoxycholate, filtered with 0.45 micron filter unit), then washed once with cold 1X TE (10 mM Tris-HCl pH 7.5 , 0.1 mM Na_2-EDTA). Bead pellets were resuspended in 200ul room-temperature IP elution buffer (1% SDS, 0.1 M $NaHCO_3$, filtered with 0.45 micron filter unit) and vortexed.

Reverse crosslinking and DNA purification. Samples were incubated in a 65C water bath for 1 hour with mixing at 15-minute intervals to uncouple beads from protein-DNA complexes. Samples were centrifuged (14000 rmp, 3 mins) and placed on a magnet to pellet beads, and supernatants were isolated then incubated in a 65C water bath overnight to reverse crosslinks. DNA was purified using a Qiagen MinElute reaction cleanup kit and quantified using a Qubit High Sensitivity DNA kit.

2.4 Sequence filtering and processing

ChIP and total chromatin DNA samples from transfected and untransfected cells were sequenced in multiplexed paired-end Illumina libraries, yielding 51bp reads. Samples from transfected cells were multiplexed across 3 lanes, yielding roughly 180M reads per sample. Samples from untransfected cells were multiplexed across 2 lanes, yielding roughly 90M reads per sample. Sequencing reads were aligned to hg19 using BWA [7], and reads not mapped in a proper pair with insert size smaller than 10kb were removed. Read pairs representing likely PCR duplicates were also removed. Fragment coverage was computed at each position in the genome and in 100bp non-overlapping bins (note: fragment coverage, as opposed to read coverage, includes all bases between two paired reads) using in-house code and the samtools and bedtools packages [8, 9].

2.5 Calling PRDM9 binding peaks

Definitions

Let $D_1(i)$, $D_2(i)$ and $G(i)$ be random variables representing the fragment coverage in a 100bp bin i from the two ChIP-seq replicates and the genomic control, respectively (and let $d_1(i)$, $d_2(i)$ and $g(i)$ represent the observed coverage in bin i). We model the coverage of each sequencing replicate j at bin i as a sample from a Poisson distribution with mean $\lambda_j(i)$,

$$\begin{aligned} D_1(i) &\sim Poisson(\lambda_1(i)), \\ D_2(i) &\sim Poisson(\lambda_2(i)), \\ G(i) &\sim Poisson(\lambda_g(i)), \end{aligned}$$

$$\begin{aligned} \lambda_1(i) &= \alpha_1 b(i) + c(i), \\ \lambda_2(i) &= \alpha_2 b(i) + \beta c(i), \\ \lambda_g(i) &= b(i), \end{aligned}$$

where α_1 and α_2 are constants defining how coverage due to background in the ChIP replicates compares to $b(i)$, a parameter representing the mean coverage in the genomic control lane at bin i; and β is a constant defining how coverage due to binding enrichment in ChIP replicate 2 compares to $c(i)$, a parameter representing the coverage due to binding enrichment in ChIP replicate 1 at bin i. We wish to test the hypothesis that $c(i) \geq 0$ for each bin i.

The Poisson distribution was chosen as a model of sequencing coverage given its support on all non-negative integers and simple parameterisation. As specified, this model assumes that the coverage due to signal is proportional between the two ChIP-seq replicates across the genome (according to the constant β) and that the coverage due to background is proportional among all 3 lanes across the genome (specified by constants α_1 and α_2). We allow for local estimates of background and signal to account for sequence coverage biases and mappability differences across the genome.

Estimating constants

One can estimate α_j by assuming (conservatively) that when $d_1(i) = 0$ or $d_2(i) = 0$, $c(i) = 0$. That is, one can assume that if ChIP replicate j has coverage 0 at bin i, then any coverage in the other replicate (j') arises purely from background. Thus for all i such that $d_j(i) = 0$

$$\begin{aligned} \lambda_{j'}(i) &= \alpha_{j'} b(i), \\ \mathbb{E}_{genome}[\lambda_{j'}(i)] &= \alpha_{j'} \mathbb{E}_{genome}[b(i)], \end{aligned}$$

and thus one can estimate $\alpha_{j'}$ as

$$\hat{\alpha}_{j'} = \frac{\sum\limits_{i:d_j(i)=0} d_{j'}(i)}{\sum\limits_{i:d_j(i)=0} g(i)}.$$

Now β can be estimated using genome-wide coverage means $\bar{d}_1$, $\bar{d}_2$, $\bar{g}$ as follows:

$$\bar{d}_1 \approx \hat{\alpha}_1 \bar{g} + \mathbb{E}_{genome}[c(i)],$$
$$\bar{d}_2 \approx \hat{\alpha}_2 \bar{g} + \beta \mathbb{E}_{genome}[c(i)],$$

$$\hat{\beta} \approx \frac{\bar{d}_2 - \hat{\alpha}_2 \bar{g}}{\bar{d}_1 - \hat{\alpha}_1 \bar{g}}.$$

Hypothesis Testing

With these estimates of α_j and β, one can compute Maximum Likelihood Estimators for the unknown parameters $b(i)$ and $c(i)$ at each bin i from the coverage data $d_1(i)$, $d_2(i)$ and $g(i)$ (see below for derivation). Then, using these MLEs one can compute a log-likelihood ratio test statistic against a null model in which $c(i) = 0$:

$$\Lambda(i) = 2\log \frac{\max\limits_{b(i),c(i)\geq 0} [L(D_1(i) = d_1(i), D_2(i) = d_2(i), G(i) = g(i))]}{\max\limits_{b(i),c(i)=0} [L(D_1(i) = d_1(i), D_2(i) = d_2(i), G(i) = g(i))]}.$$

Under the null hypothesis, the test statistic $\Lambda(i)$ is distributed approximately as a χ^2 distribution (with 1 degree of freedom due to the parameter $c(i)$ and an atom of probability at 0), yielding a p-value at each bin i indicating the probability that the observed likelihood ratio could arise from background alone.

Calculation of Maximum Likelihood Estimators

Recall that at each position the Poisson means for coverage in each lane are (dropping the i notation for succinctness)

$$\lambda_1 = \hat{\alpha}_1 b + c,$$
$$\lambda_2 = \hat{\alpha}_2 b + \hat{\beta} c,$$
$$\lambda_g = b,$$

where $\hat{\alpha_1}$, $\hat{\alpha_2}$, and $\hat{\beta}$ are constants estimated for the whole genome. To simplify calculations, we reparameterise using a new variable $y = c/b$ and rewrite the above equations as

$$\begin{aligned}
\lambda_1 &= \hat{\alpha_1} b + yb, \\
\lambda_2 &= \hat{\alpha_2} b + \hat{\beta} yb, \\
\lambda_g &= b.
\end{aligned}$$

Given the observed coverage values d_1, d_2, and g, the Poisson log likelihood function can be written as

$$\begin{aligned}
\ell &\propto -\lambda_1 + d_1 \log(\lambda_1) - \lambda_2 + d_2 \log(\lambda_2) - \lambda_g + g\log(\lambda_g) \\
&= -\hat{\alpha_1} b - yb + d_1 \log(\hat{\alpha_1} b + yb) - \hat{\alpha_2} b - \hat{\beta} yb + d_2 \log(\hat{\alpha_2} b + \hat{\beta} yb) - b + g\log(b) \\
&= -b(\hat{\alpha_1} + \hat{\alpha_2} + 1) - yb(1 + \hat{\beta}) + d_1 \log(\hat{\alpha_1} b + yb) + d_2 \log(\hat{\alpha_2} b + \hat{\beta} yb) + g\log(b). \qquad (1)
\end{aligned}$$

Now to maximise ℓ we first obtain the partial derivatives for b and y

$$\begin{aligned}
\frac{\partial \ell}{\partial b} &= -(\hat{\alpha_1} + \hat{\alpha_2} + 1) - y(1 + \hat{\beta}) + \frac{d_1(\hat{\alpha_1} + y)}{b(\hat{\alpha_1} + y)} + \frac{d_2(\hat{\alpha_2} + \hat{\beta} y)}{b(\hat{\alpha_2} + \hat{\beta} y)} + \frac{g}{b} \\
&= -(\hat{\alpha_1} + \hat{\alpha_2} + 1) - y(1 + \hat{\beta}) + \frac{1}{b}(d_1 + d_2 + g), \qquad (2) \\
\frac{\partial \ell}{\partial y} &= -b(1 + \hat{\beta}) + \frac{d_1 b}{b(\hat{\alpha_1} + y)} + \frac{d_2 \hat{\beta} b}{b(\hat{\alpha_2} + \hat{\beta} y)} \\
&= -b(1 + \hat{\beta}) + \frac{d_1}{(\hat{\alpha_1} + y)} + \frac{d_2 \hat{\beta}}{(\hat{\alpha_2} + \hat{\beta} y)}. \qquad (3)
\end{aligned}$$

Next, we set the partials to 0 and solve them as a system to obtain any potential local maxima. We start by solving for b in Equation 2 as follows:

$$\begin{aligned}
0 &= -(\hat{\alpha_1} + \hat{\alpha_2} + 1) - y(1 + \hat{\beta}) + \frac{1}{b}(d_1 + d_2 + g); \\
b &= \frac{d_1 + d_2 + g}{\hat{\alpha_1} + \hat{\alpha_2} + 1 + y(1 + \hat{\beta})}. \qquad (4)
\end{aligned}$$

Then, we substitute it into Equation 3 and rewrite it as follows, with the aim of simplifying

it into quadratic form:

$$
\begin{aligned}
0 &= -\frac{d_1 + d_2 + g}{\hat{\alpha_1} + \hat{\alpha_2} + 1 + y(1+\hat{\beta})}(1+\hat{\beta}) + \frac{d_1}{(\hat{\alpha_1} + y)} + \frac{d_2\hat{\beta}}{(\hat{\alpha_2} + \hat{\beta}y)}; \\
\frac{(d_1 + d_2 + g)(1+\hat{\beta})}{\hat{\alpha_1} + \hat{\alpha_2} + 1 + y(1+\hat{\beta})} &= \frac{d_1(\hat{\alpha_2} + \hat{\beta}y) + d_2\hat{\beta}(\hat{\alpha_1} + y)}{(\hat{\alpha_1} + y)(\hat{\alpha_2} + \hat{\beta}y)} \\
&= \frac{d_1\hat{\alpha_2} + d_1\hat{\beta}y + d_2\hat{\beta}\hat{\alpha_1} + d_2\hat{\beta}y}{\hat{\alpha_1}\hat{\alpha_2} + \hat{\alpha_1}\hat{\beta}y + \hat{\alpha_2}y + \hat{\beta}y^2} \\
&= \frac{y(d_1\hat{\beta} + d_2\hat{\beta}) + d_1\hat{\alpha_2} + d_2\hat{\beta}\hat{\alpha_1}}{\hat{\alpha_1}\hat{\alpha_2} + y(\hat{\alpha_1}\hat{\beta} + \hat{\alpha_2}) + \hat{\beta}y^2}. \qquad (5)
\end{aligned}
$$

To shorten notation, we substitute in the following variables for constant terms in Equation 5:

$$
\begin{aligned}
t_1 &= (g + d_1 + d_2)(1+\hat{\beta}), \\
t_2 &= \hat{\alpha_1} + \hat{\alpha_2} + 1, \\
t_3 &= 1 + \hat{\beta}, \\
t_4 &= d_1\hat{\alpha_2} + d_2\hat{\beta}\hat{\alpha_1}, \\
t_5 &= d_1\hat{\beta} + d_2\hat{\beta}, \\
t_6 &= \hat{\alpha_1}\hat{\alpha_2}, \\
t_7 &= \hat{\alpha_1}\hat{\beta} + \hat{\alpha_2},
\end{aligned}
$$

yielding

$$
\begin{aligned}
\frac{t_1}{t_2 + yt_3} &= \frac{yt_5 + t_4}{t_6 + yt_7 + \hat{\beta}y^2}; \\
0 &= t_1(t_6 + yt_7 + \hat{\beta}y^2) - (t_2 + yt_3)(yt_5 + t_4); \\
0 &= t_1t_6 + yt_1t_7 + t_1\hat{\beta}y^2 - yt_2t_5 - t_2t_4 - y^2t_3t_5 - yt_3t_4; \\
0 &= y^2(t_1\hat{\beta} - t_3t_5) + y(t_1t_7 - t_2t_5 - t_3t_4) + (t_1t_6 - t_2t_4). \qquad (6)
\end{aligned}
$$

Now we can solve for y in Equation 6 using the quadratic formula, taking the positive root to be $\hat{y}$, the MLE for y. To obtain $\hat{b}$, we simply substitute $\hat{y}$ into Equation 4. Finally, to obtain $\hat{b}_0$, the MLE for b under the background model, we can simply set y to 0 in Equation 4, yielding

$$
\hat{b}_0 = \frac{d_1 + d_2 + g}{\hat{\alpha_1} + \hat{\alpha_2} + 1}.
$$

Peak calling and centring

We identified all contiguous regions whose 100bp bins had $p < 10^{-5}$ and then repeated likelihood ratio testing at each individual base in those regions. Within each of these regions

we identified the position with the largest likelihood ratio, taking this to be the centre of the underlying binding peak. We then defined peak boundaries as the nearest positions to the left and right where $\Lambda(i)$ drops by at least 9.12 (but no closer than 50bp to the centre), thus defining a 99% confidence interval (using χ^2_2, with one df for the enrichment factor and one for the hotspot centre position) likely to contain the true binding site. Finally, we merged overlapping intervals to yield our final set of peak regions.

PAR1 peaks

The locations of all ChIP-seq binding peaks in PAR1 are provided in Dataset S5.

References

[1] Hinch AG, Tandon A, Patterson N, Song Y, Rohland N, et al. (2011) The landscape of recombination in African Americans. Nature 476: 170–175.

[2] Lander ES, Green P (1987) Construction of multilocus genetic linkage maps in humans. Proc Natl Acad Sci U S A 84: 2363–2367.

[3] Rabiner L, Juang B (1986) An introduction to hidden Markov models. IEEE ASSP Mag 3: 4–16.

[4] Hinch A (2013) The landscape of recombination in African Americans. Ph.D. thesis, Oxford University Research Archive.

[5] Aricescu AR, Lu W, Jones EY (2006) A time- and cost-efficient system for high-level protein production in mammalian cells. Acta Crystallogr D Biol Crystallogr 62: 1243–1250.

[6] Johnson DS, Mortazavi A, Myers RM, Wold B (2007) Genome-wide mapping of in vivo protein-DNA interactions. Science 316: 1497–1502.

[7] Li H, Durbin R (2009) Fast and accurate short read alignment with Burrows-Wheeler transform. Bioinformatics 25: 1754–1760.

[8] Quinlan AR, Hall IM (2010) BEDTools: a flexible suite of utilities for comparing genomic features. Bioinformatics 26: 841–842.

[9] Li H, Handsaker B, Wysoker A, Fennell T, Ruan J, et al. (2009) The Sequence Alignment/Map format and SAMtools. Bioinformatics 25: 2078–2079.
